# Efficient parallelization of tensor network contraction for simulating quantum computation

**DOI:** 10.1038/s43588-021-00119-7

**Published:** 2021-09-13

**Authors:** Cupjin Huang, Fang Zhang, Michael Newman, Xiaotong Ni, Dawei Ding, Junjie Cai, Xun Gao, Tenghui Wang, Feng Wu, Gengyan Zhang, Hsiang-Sheng Ku, Zhengxiong Tian, Junyin Wu, Haihong Xu, Huanjun Yu, Bo Yuan, Mario Szegedy, Yaoyun Shi, Hui-Hai Zhao, Chunqing Deng, Jianxin Chen

**Affiliations:** 1 Alibaba Quantum Laboratory, Alibaba Group USA, Bellevue, WA USA; 2 Department of Electrical Engineering and Computer Science, University of Michigan, Ann Arbor, MI USA; 3 Departments of Physics and Electrical and Computer Engineering, Duke University, Durham, NC USA; 4 Alibaba Quantum Laboratory, Alibaba Group, Hangzhou, Zhejiang China; 5 Alibaba Cloud Intelligence, Alibaba Group USA, Bellevue, WA USA; 6 Alibaba Cloud Intelligence, Alibaba Group, Hangzhou, Zhejiang China; 7 Alibaba Quantum Laboratory, Alibaba Group, Beijing, Beijing China

**Keywords:** Quantum information, Computational science

## Abstract

We develop an algorithmic framework for contracting tensor networks and demonstrate its power by classically simulating quantum computation of sizes previously deemed out of reach. Our main contribution, index slicing, is a method that efficiently parallelizes the contraction by breaking it down into much smaller and identically structured subtasks, which can then be executed in parallel without dependencies. We benchmark our algorithm on a class of random quantum circuits, achieving greater than 10^5^ times acceleration over the original estimate of the simulation cost. We then demonstrate applications of the simulation framework for aiding the development of quantum algorithms and quantum error correction. As tensor networks are widely used in computational science, our simulation framework may find further applications.

## Abstract (web summary)

An efficient method for parallelizing the contraction of tensor networks pushes the boundaries for the classical simulation of quantum computation, and aids the development of quantum algorithms and hardware.

## Main

Quantum computers offer the promise of exponential speedups over classical computers. Consequently, as quantum technologies grow in scale, there will be an inevitable crossing point after which nascent quantum processors will overtake behemoth classical computing systems in performing specialized tasks. The term quantum supremacy was coined to describe this watershed moment^1^, which we refer to in this paper as quantum superiority. Recent advances in quantum computing hardware have resulted in quantum processors with more than 50 qubits, and there have been multiple claims that quantum superiority has been achieved on different quantum devices, including superconducting systems^2^ and photonic quantum devices^3^. These milestones mark the start of the era of noisy intermediate-sized quantum devices^4^.

With quantum devices increasing in size and precision, classical simulation of the corresponding quantum systems also becomes increasingly challenging. Classical simulation plays an indispensable role in understanding and designing quantum devices, as it is often, if not always, the only means to validate and benchmark existing quantum devices. Although there have already been numerous efforts in designing and implementing efficient classical simulators^5–7^, there is always a push to simulate larger quantum devices. The reason is twofold: first, simulating large quantum systems helps reduce the finite-size effect observed in certain experiments with smaller quantum systems, which allows us to more confidently project the performance of large quantum systems in which classical simulation is definitely out of reach. Second, claims of quantum superiority are based on the assumption that classical simulation cannot achieve a task that is easily achievable using quantum devices. This is not sound without an extensive effort to push the boundaries of classical simulability.

In this paper we propose a highly optimized framework for classically simulating intermediate- to large-scale quantum computations, represented as tensor networks. Tensor network contraction has been one of the prominent choices for simulating quantum computation due to its high flexibility and expressive power; however, exact contraction of general tensor networks is a computationally hard problem with respect to the problem size: there are tensor networks for which an exact simulation would take exponential amount of time under well-established computational complexity assumptions^8,9^. Nevertheless, exact tensor network contraction is indispensable in cases in which a numerically accurate approximation ansatz has not yet been established, suffers from practical inefficiency (for example, repeated singular value decomposition) or outputs poor-quality results. In reality, most tensor network instances of interest are far from the worst-case scenario and the contraction efficiency can have orders of magnitude improvements compared with the naive approach by optimizing the contraction procedure. This is the focus of our paper.

In addition to developing and conglomerating several technical optimizations for tensor network contraction, the main technical contribution of our paper is a framework to parallelize tensor network contraction called index slicing. Index slicing decomposes a tensor network contraction task into many subtasks that have identical shapes and can be executed in an embarrassingly parallel way, that is, there is no dependency or communication required between the execution of the subtasks. Such an algorithm can be readily deployed on modern computational clusters and experimental evidence shows that such parallelization introduces little overhead to the total running time. As tensor networks are ubiquitous in quantum information science (with applications including benchmarking quantum devices^2^, probing quantum many-body systems^10–13^ and decoding quantum error-correcting codes^14–17^), our simulator represents a useful tool to aid in the development of quantum technologies.

One major challenge of index slicing is controlling the overhead introduced to the total running time. This overhead is usually not noticeable when the number of indices needed to be sliced is very small, but it can quickly grow out of control for a larger number of sliced indices. Multiple works have been dedicated to addressing this problem^18–21^. In this paper we develop a heuristic algorithm to minimize the overhead by interleaving finding the best index to slice with local optimization of the contraction order. To further improve performance, we focus the local optimizations on tensor network contraction steps that takes the most amount of time and space, allowing more rounds of local optimization.

As a benchmarking example, we test our algorithm on the simulation task that prompted the quantum-superiority claim made in Arute and co-workers^2^. This task—called Sycamore random circuit sampling—is to output bitstrings distributed according to the measurements of random quantum circuits that are designed to be executed on Google’s recent 53-qubit Sycamore device. This task is considered relatively easy on the Sycamore quantum chip, while at the same time infeasibly hard for any classical computational device. It is estimated that such a sampling task takes about 200 s to achieve on the Sycamore quantum chip, but would take over 10,000 years for the then-best supercomputer Summit^2^. By experimenting on a subsample of the task on the GPU cluster at Alibaba, we show that our embarrassingly parallel tensor network contraction algorithm can indeed be carried out without extra cost, and it can complete the random circuit sampling task within 20 days on a Summit-comparable cluster. Furthermore, to demonstrate the usefulness and broad capabilities of the tensor network-based simulation framework, we apply it to both the studies of near-term quantum algorithms and fault-tolerant quantum computing. The two examples we studied are the quantum approximate optimization algorithm (QAOA) as a candidate for graph isomorphism discovery, and the performance of the Surface-17 in a quantum memory experiment under noise models including neighbouring qubit stray ZZ-interaction. In both cases, the simulation tasks go slightly beyond quantum circuits, but they fall easily into the grasp of our simulation framework, indicating flexibility of our framework in the area of quantum computing.

## Results

### Efficient contraction of tensor networks

The tensor network is a well-studied framework for expressing multilinear functions over multidimensional arrays called tensors, and it is extensively used in multiple areas including quantum physics^22,23^, machine learning^24,25^ and quantum computation^26,27^. A tensor network can be formulated as a mutlihypergraph. Each node of a tensor network is associated with a tensor and each of its connecting edges corresponds to one dimension of the tensor. A hyperedge in a tensor network can connect multiple tensor nodes, indicating that the corresponding dimensions are identified. Furthermore, a hyperedge is either closed or open. The computational task associated with a tensor network—called the tensor network contraction—is to compute an output tensor given the values of the tensor nodes and the hypergraph structure. Each dimension of the output tensor corresponds to an open edge in the tensor network. A particular entry in the output tensor corresponds to an assignment of the open edges, and its value equals the summation over all possible assignments of the closed edges of the product of the corresponding entries of the input tensors.

#### Sequential pairwise contraction

One common method for exactly contracting tensor networks is through sequential pairwise contraction. In each step, two tensors from the tensor network are selected and merged together according to their shared indices, in a way similar to matrix multiplication. This reduces the number of tensor nodes in the tensor network by one. By repeatedly applying the pairwise contraction, one is left with a single tensor in the tensor network at the end, which is the result of the tensor network contraction. A cleverly chosen order of pairwise contraction can often reduce the total time complexity of tensor network contraction by several orders of magnitude, making it one of the most time-efficient tensor network contraction algorithms. Over the years many heuristics have been proposed to find efficient sequential pairwise contraction orders^26–29^; however, sequential pairwise contraction suffers from intrinsic sequentiality and a sometimes inevitable space complexity lower bound; both greatly affect the scalability of such algorithms.

#### Index slicing

To remedy these two problems, we propose a parallelization framework called index slicing that aims to divide a tensor network contraction task into many subtasks with identical tensor network structures such that the subtasks can be executed in parallel, each with a space complexity small enough to fit into a single computational unit. Index slicing starts by selecting a subset of the hypergraph indices. Each subtask then corresponds with a partial sum where the assignment of the selected sliced indices are fixed; it is itself a tensor network. The subtask tensor networks often have simpler structures, allowing them to be contracted sequentially pairwise with reduced space complexity. A contraction of the whole tensor network is then reduced to summing up the partial results obtained from the contractions of the individual subtasks.

**Fig. 1:**
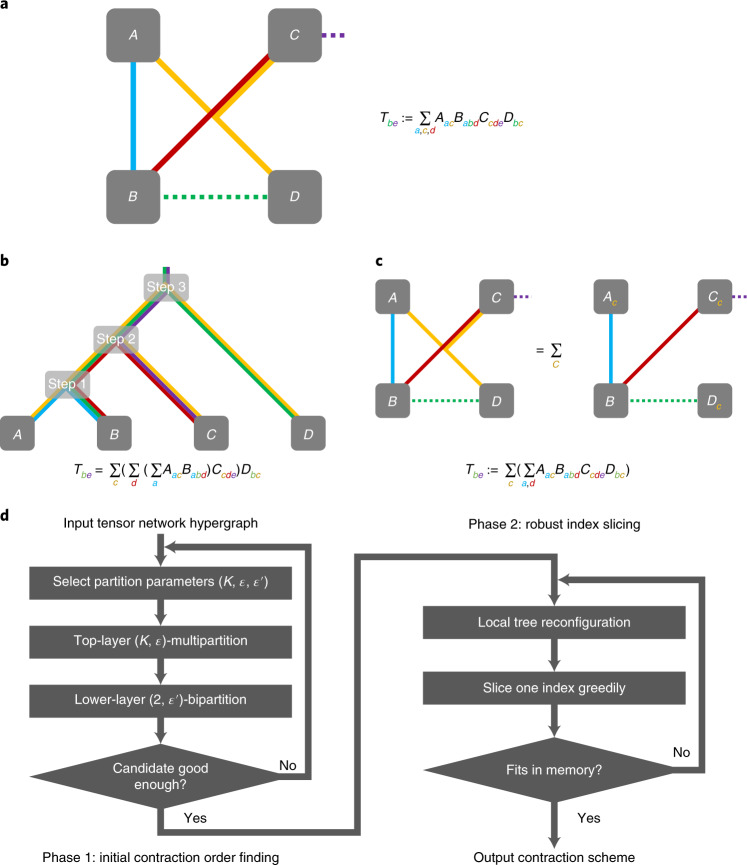
An illustration of tensor networks, sequential pairwise contraction, index slicing and the contraction scheme-finding heuristics. **a**, An example of a tensor network with four tensor nodes and five edges, where the edges *a*, *c*, *d* are closed and *b*, *e* are open. **b**, A sequential contraction order, where two tensors are merged into one at each step. The edges going upwards indicate the corresponding indices in the intermediate results. **c**, A slicing of the index *c*, resulting in identically structured tensor networks labelled by all possible values of *c*. The subtensor networks are to be contracted individually and summed up at the end. **d**, A two-phase heuristic used to find a good contraction order and index slicing for a given tensor network structure, which will be discussed in detail in the Methods.

In practice, memory constraints are far more rigid than running time constraints, and many modern computer architectures allow for massive parallelism. Index slicing effectively makes use of the latter while coping with the former, thereby improving on previous tensor network contraction algorithms both in efficiency and scalability. Of course, selecting the best subset of indices to slice over is a non-deterministic polynomial-time (NP)-hard optimization problem, for which no known algorithm is guaranteed to find an optimal solution in polynomial time. Furthermore, it is often intertwined with the other NP-hard optimization problem of finding the best sequential contraction algorithm; however, with the heuristics discussed in the Methods, index slicing can be carried out with extremely low parallelization overhead while reducing the space complexity to a single computational node. Figure 1 illustrates the idea of tensor networks, contraction orders, index slicing and a flowchart briefing our heuristic strategies for finding good contraction orders and indices to slice.

**Fig. 2:**
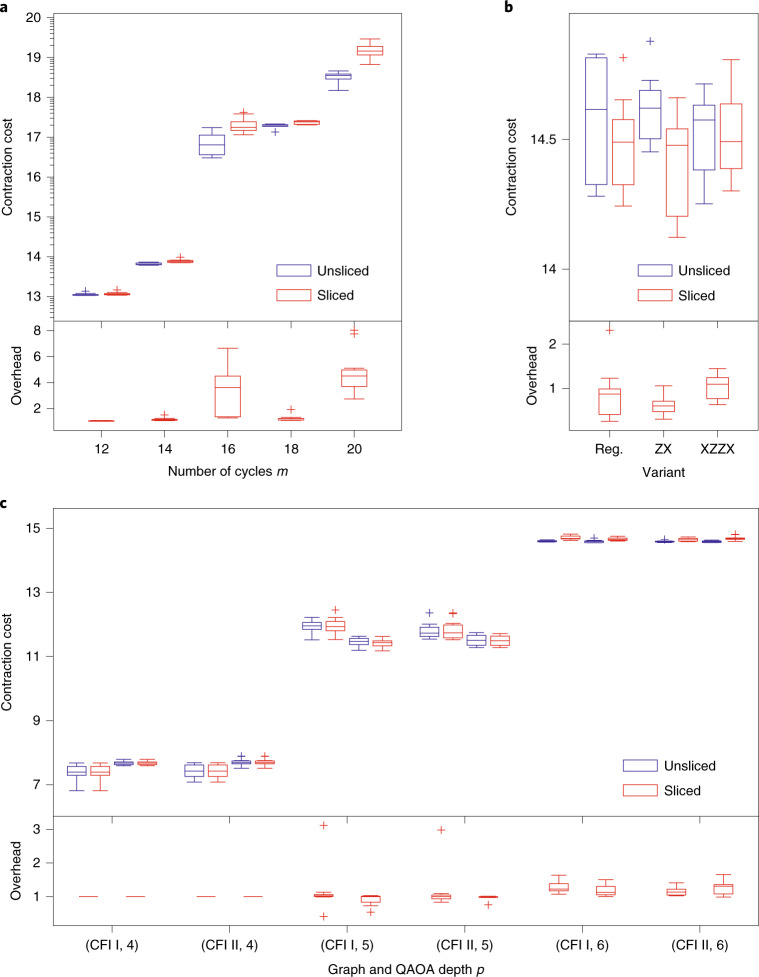
Unsliced costs, sliced costs and slicing overheads for various tensor networks studied in this paper. Each box represents the lower (Q1) to upper (Q3) quartiles of contraction costs over ten independent runs of the algorithm, with a horizontal line that represents the median. The whiskers indicate the highest and lowest contraction costs/overheads that are not outliers, where outliers are defined as data points whose distance to the nearest quartile is larger than 1.5 times the interquartile range. **a**, Tensor networks for evaluating a batch of 64 amplitudes in Sycamore random circuits. **b**, Tensor networks corresponding to 2 + 1 rounds of syndrome extraction for the Surface-17 code. **c**, Tensor networks associated with edges of the Cai–Fürer–Immerman (CFI) graphs. For each graph and each QAOA depth, there are two pairs of unsliced/sliced costs for the two isomorphic classes of edges in that graph: the left pair corresponds to the first class, whereas the right pair corresponds to the second class (see Fig. 4).

Unless specifically indicated, all indices in the tensor network run through {0, 1}, a common assumption for qubit-based quantum computation. Throughout the results we report the complexity of contracting different tensor networks by a pair of numbers: the first one, called the computation cost, is the base-ten logarithm of the number of total floating point operations (FLOPS), serving as a measure for the time complexity. The second one, the number of subtasks, is exponential in the number of sliced indices, and serves as a measure of concurrency for the contraction algorithm. Moreover, we measure the quality of the index slicing by the slicing overhead, that is, the ratio of the total time complexity before and after performing index slicing. Figure 2 summarizes the contraction costs and slicing overheads for various tensor networks studied in this paper.

### Classical simulation of Sycamore random circuit sampling

We first benchmark the open-source implementation of our simulation framework (the Alibaba Cloud Quantum Development Platform, ACQDP) with a family of circuits called the Sycamore random circuits, which were originally proposed to demonstrate quantum superiority^2^. It was claimed that when the number of layers *m* in the circuit is 20, a certain sampling task could be efficiently performed on the existing Sycamore quantum chip in about 200 s, whereas a comparable task would take Summit—one of the most powerful supercomputers in the world—at least 10,000 years.

We measure the performance of various simulation frameworks based on tensor network contraction with the contraction cost and an extrapolated running time that is based on actually running some of the many structurally identical subtasks created by index slicing. The ACQDP achieves an exceptionally low contraction cost—up to 10^6^-times lower than qFlex^21^ and up to 1,000-times lower than Cotengra^28^; however, the FLOPS efficiency of ACQDP is also considerably lower than that of Cotengra and qFlex. This is probably due to the involvement of many general matrix–matrix products with small-sized matrices during the computation. Overall, ACQDP forecasts a running time of less than 20 days for the *m* = 20 random circuit sampling task on a Summit-comparable computing cluster, a speedup of more than five orders of magnitude when compared with the best classical algorithms reported by Arute and colleagues^2^, and a speedup of more than two orders of magnitude when compared with other state-of-the-art simulators.

**Fig. 3:**
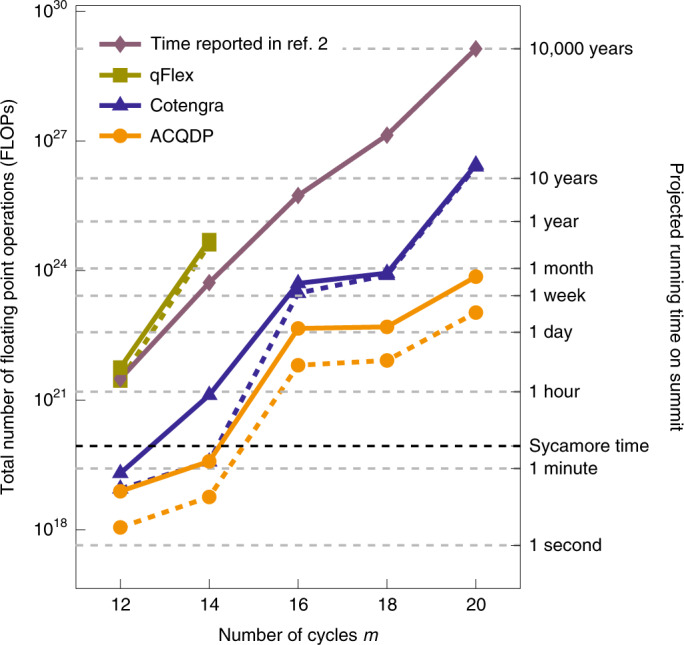
Classical simulation cost and extrapolated running time of sampling from *m*-cycle Sycamore random circuits with low XEB fidelities. The dashed lines represent the theoretical number of FLOPs and the solid lines represent extrapolated running times from the experiments on an Nvidia V100 graphics card. The two axes are aligned by the theoretical GPU efficiency of a Nvidia V100. Consequently, the dashed lines represent runtime lower bounds provided that GPU efficiency is fully saturated. The velvet line is reported in Arute and colleagues^2^ using the hybrid Schrödinger–Feynman algorithm.

The contraction cost, FLOPS efficiency, extrapolated runtime and comparisons with other leading simulators are all illustrated in Fig. 3. For all of the tensor network-based simulators (qFlex, Cotengra and ACQDP), a batch of amplitudes is computed using open tensor network contraction. Due to randomness in the ACQDP, we ran ten independent order-finding experiments for each number of cycles *m* (the statistical results are presented in Fig. 2a). The orders found show good concentration in time complexity, and we take the best orders found for the comparisons reported in Fig. 3. The projected running time of the hybrid Schrödinger–Feynman algorithm reported in ref. ^2^ is estimated from a different architecture than Summit, and so the FLOPs efficiency is not shown.

A very recent result by Pan and colleagues^30^ claimed to have completed the Sycamore random circuit sampling task by producing one million distinct but correlated bitstrings within five days using a small GPU cluster, achieving a linear cross-entropy benchmarking fidelity (XEB) value of ~0.739. This is made possible by combining hierarchical partitioning and dynamic index slicing of tensor networks with their newly developed heuristics. Although their work verifiably passes the linear XEB test, it essentially completes a different task than what we describe as an unbiased-noise approximate (UNA) sampling. A detailed discussion of the definition of the random circuit sampling task is presented in Supplementary Section 3C.

### QAOA for graph isomorphism discovery

We investigate a potential application of the QAOA, which is to determine whether two graphs are isomorphic by checking whether their QAOA energy functions are equal^31^. It is not clear whether this method can distinguish between all pairs of non-isomorphic graphs (for sufficiently large number of QAOA layers *p*) or whether the energy gap would be noticeable. Here we try to study these questions by using ACQDP to classically compute QAOA energies associated with various graphs.

By classically computing the QAOA energies, we can separate all non-isomorphic 3-regular graphs up to size 18, all strongly regular graphs up to size 26, and several hard graph pairs including the Miyazaki and Praust graphs of size 20, and the Cai–Fürer–Immerman graphs of size 40. These findings and the theoretical results in Szegedy^31^ make us believe that QAOA energies give a full characterization of isomorphism classes, unlike many quantum walk-based distinguishers that were considered earlier^32–34^. Table 1 provides pairs or classes of graphs, as well as the QAOA depth *p* that distinguish them.

**Table 1:** Summary of results for using the QAOA to distinguish non-isomorphic graph sets.

Class or pair of graphs	Number of nodes	QAOA depth giving full separation	Contraction cost
Miyazaki I and II	20	4	10.1
Praust I and II	20	4	10.5
Cai–Fürer–Immerman graphs I and II	40	6	15.4
All 4,060 non-isomorphic 3-regular graphs on 16 nodes^51^	16	4	8.7
All 41,301 non-isomorphic 3-regular graphs on 18 nodes^51^	18	4	9.3
All 10 non-isomorphic graphs in the SRG 26,10,3,4 family^52^	26	3	12.8

Experiments were conducted on the 20-node Miyazaki graphs, 20-node Praust graphs, 40-node Cai–Fürer–Immerman graphs, all non-isomorphic 3-regular graphs on 16 and 18 nodes, and all non-isomorphic (26, 10, 3, 4) strongly regular graphs. The number of nodes, the minimum QAOA depth to tell all of the graphs apart, and the average contraction cost for one graph, are listed. Only the Cai–Fürer–Immerman graph pair requires index slicing, as the graphs contain many nodes and it takes a deep QAOA circuit to tell the two graphs apart.

**Fig. 4:**
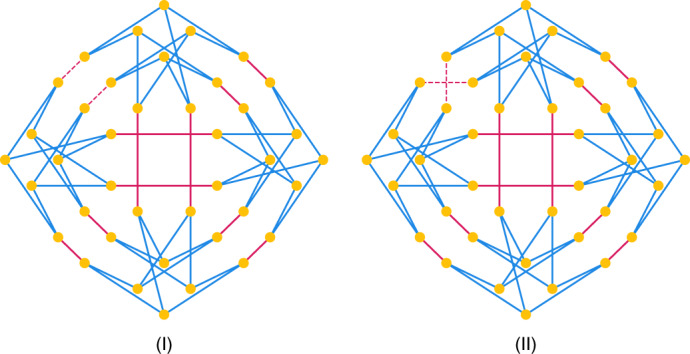
Cai–Fürer–Immerman graphs I and II with 40 indices. For each graph there are two isomorphic classes of edges, with the first class colored blue and the second class colored red. Note that the only difference between the two graphs is the two red edges in the upper left corner.

Our simulator can cope with Cai–Fürer–Immerman graphs of size 40, a well-known pair of hard instances (see Fig. 4). This instance is hard even for the QAOA due to the fact that the two graphs cannot be distinguished until the QAOA depth reaches six. In fact, Fig. 2c shows the contraction cost of the QAOA instances. It is worth noting that most of the slicing induces extremely low slicing overhead. The overall average overhead introduced by one slice for all of the *p* = 5 and *p* = 6 tensor networks is 0.2% and 3.5%, respectively.

### Simulating surface codes with cross-talk errors

We simulate a quantum memory experiment on a surface code with 17 qubits—Surface-17 for short—in the presence of a practical noise model^35^ and a ZZ cross-talk model (see Methods), which was not considered before in this context. The performance of the surface code is measured by the Pauli transfer matrix (PTM) on the logical qubit.

#### Effects of cross-talk for 2 + 1 rounds of syndrome extraction

To compute the logical PTM under the optimal decoder, one needs to compute all of the PTMs corresponding to quantum operations for each assignment of the syndrome bits. As mentioned before, our tensor network-based approach can deal with up to 2 + 1 rounds of syndrome extraction, as this would result in a resulting tensor of size 2^28^. Note that this is also the depth proposed for a near-term fault-tolerance demonstration^36^.

**Fig. 5:**
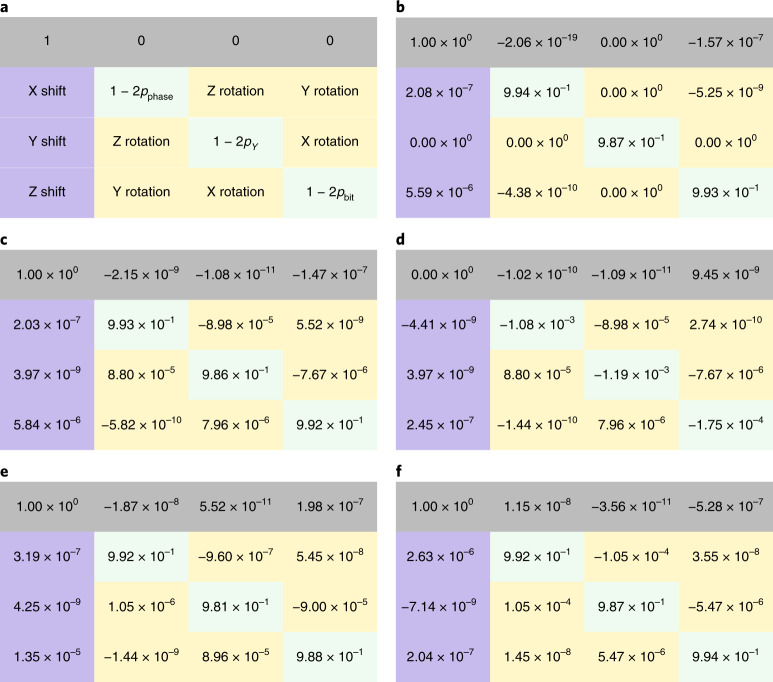
Comparisons of logical channels with and without cross-talk for 2 + 1 rounds of syndrome extraction. **a**, An illustration of a PTM corresponding to a single-qubit completely positive and trace-preserving map. Mathematically, for all completely positive and trace-preserving maps, the first row of the corresponding PTMs should be (1, 0, 0, 0). We report the computational result from the tensor network contraction on an Nvidia V100 graphics card, and use the deviation of the first row to (1, 0, 0, 0) to indicate the magnitude of the numerical imprecision. **b**, Logical PTM for the default variant for 2 + 1 rounds of syndrome extraction without cross-talk. **c**, Logical PTM for the default variant for 2 + 1 rounds of syndrome extraction with cross-talk. **d**, The difference between the two logical PTMs in **b** and **c**. **e**, Logical PTM for 2 + 1 rounds of syndrome extraction with cross-talk, *Z*/*X* switched. **f**, Logical PTM for 2 + 1 rounds of syndrome extraction with cross-talk, XZZX variant.

We report the logical channels for 2 + 1 rounds of syndrome extraction, with and without the presence of cross-talk (Fig. 5). We can infer from the table that the effect of ZZ cross-talk on the logical channel is concentrated on the logical coherent *Z*-rotation and the stochastic phase-flip error, each ~10^−3^ in magnitude. Compared with other error sources, ZZ cross-talk introduces a minimal amount of logical error and thus does not present itself as a main error source for the quantum memory experiment.

#### Results with variants in code and gate scheduling

We test the performance of slight variants of the above gate scheduling on Surface-17 to validate the robustness of our findings. We first switch the *Z*-stabilizer syndrome extractions and the *X*-stabilizer ones in each cycle to see whether considerable changes in logical errors can be observed. We then test out the recently proposed XZZX code^37^ to balance the bias in *X*- and *Z*- Pauli errors. The corresponding PTMs are listed in Fig. 5.

For the experiment switching *X*- and *Z*- syndrome extraction, it can be observed that the *X*- and *Z*- portions of the logical errors are also switched. This agrees with the observation that such a error correction circuit is similar to one where a transversal Hadamard gate is applied to all of the physical qubits, resulting in a logical Hadamard gate interchanging the *X*- and *Z*- Pauli errors. On the other hand, merely changing the code to its XZZX variant does not balance the logical noises. This is probably due to the fact that the majority of the error occurs during the syndrome extraction routines in this particular experiment, not between them. Although XZZX variants are good at averaging biases occurring outside of the error correction cycles, it does not introduce much difference during the actual syndrome extraction routines.

#### Tensor network contraction cost

Figure 2b shows the contraction cost of the tensor networks corresponding to the experiments on different variants of the Surface-17. One particularly interesting phenomenon is that the slicing overhead of the surface code simulation is below one in some cases. This indicates that the initial contraction tree found by hypergraph decomposition framework is suboptimal, which could be due to the fact that the tensor networks considered here have many open edges, unlike the instances in the random circuit sampling problem or the QAOA experiments. Whether such a phenomenon exists in other tensor networks with many open edges, and whether index slicing can be applied to aid the contraction order finding in a more general setting is worth further investigation. We leave this to future work.

## Discussion

The index slicing framework proposed in our paper establishes an interpolation between the sequential pairwise contraction and the Feynman path integral algorithm, which correspond to the cases in which no index is sliced and in which all indices are sliced, respectively. For a tensor network with *m* indices associated with a hypergraph with tree width *t* and contraction width *c*, the sequential pairwise contraction achieves a time complexity of O^*^(2^*t*^), whereas the space complexity is lower bounded by Ω^*^(2^*c*^) (although not necessarily simultaneously achievable). The Feynman path integral, on the other hand, has a space complexity of *O*(*m*), yet the time complexity is Ω^*^(2^*m*^); however, how the slicing-incorporated idea interpolates between the aforementioned two extreme points requires further investigation. It also remains open whether there exists a tensor network contraction algorithm that achieves both the relatively low time complexity of O^*^(2^*t*^) and the space complexity of *O*(*m*), and it does not seem likely that a slicing-incorporated sequential pairwise contraction could achieve this limit.

On the practical side, there are still many ways to improve the performance of the tensor network contraction algorithm, potentially by several orders of magnitude. A better contraction order—together with index slicing—might be found through algorithmic refinements; however, it is known^8^ that exact contraction of general tensor networks is a #P-hard problem, a computational complexity category for which no known efficient (subexponential time) algorithms exist. It is therefore worth investigating approximation proposals such as tensor network contraction based on matrix product states^13,22,38,39^, matrix product operators^40–42^ and other such ansatzes. Such approximation proposals do not necessarily suffer from #P-hardness and could offer a big leap in simulability assuming the ansatzes are good. We leave investigation and design of efficient and relatively accurate approximate tensor network contraction methods to future work.

It has been noted that the good contraction schemes found by our algorithm, usually with relatively low time complexity, might not necessarily perform well on modern computational architectures. In fact, the 20-cycle random quantum circuit simulation indicates a very low FLOPS efficiency (~15%). This is probably due to the fact that, despite being efficient on paper, many of the contraction schemes found by our algorithm involve matrix multiplication of skewed shapes, which cannot be processed as efficiently as matrix multiplications of square matrices. A computational cost that is tailored to better reflect modern architecture design could be useful to guide better designs of contraction schemes in practice. We leave this to future work.

For these reasons, we expect that further improvements on both the algorithmic and engineering considerations can considerably reduce the overall simulation costs we report. Given the ubiquity of tensor networks in quantum information science and the efficiency of our simulator, we believe that it could provide a valuable tool in the development of quantum information technologies while helping to define the quantum superiority frontier.

## Methods

### Tensor networks contraction algorithms

#### Framework for tensor network contraction

We use index-slicing-incorporated sequential pairwise contraction to contract tensor networks. Finding the optimal contraction scheme (that is, a subset of indices to slice over and a sequential pairwise contraction order for the subtasks; identical in structure) is NP-hard; however, for large instances of tensor networks presented in this paper, a preprocessing heuristic finding near-optimal contraction schemes is often worthwhile as it makes a big difference in time/space complexities for the actual contraction task that considerably dwarfs the relatively short extra time spent on such preprocessing.

Finding an optimal contraction order and finding indices to slice are two strongly coupled optimization problems. On the one hand, good index slicing is often based on an existing contraction order, as the changes in time/space complexities caused by slicing then become manifest. On the other hand, the optimal contraction order also depends on the slicing, as the hypergraph structure is changed when some edges are removed. We therefore propose a two-phase contraction scheme-finding heuristic: in the first phase, we find a good contraction order for the unsliced tensor network. In the second phase, we look for indices to slice, interleaving index slicing with local reconfigurations of the contraction order to keep it near-optimal given the already sliced indices.

#### Initial contraction order finding

Disregarding the relative ordering between steps without data dependence, a contraction order can be regarded as a binary tree, where the leaf nodes correspond to the input tensors and the root to the final output tensor (recall the example in Fig. 1). We apply a slight augmentation of the hypergraph-decomposition-based contraction tree construction method in Gray and Kourtis^28^. Such an algorithm constructs a contraction tree top-down, by first decomposing the hypergraph into two or more components. The components are then regarded as tensor networks that are to be contracted individually and then contracted together; in other words, such a hypergraph decomposition fixes the top layers of the contraction tree. The subgraphs are dealt with in a recursive manner, until the number of nodes in a certain subgraph is small enough to allow efficient subtree constructions.

A hypergraph decomposition algorithm takes two parameters (*K*, *ϵ*) and a hypergraph, and outputs *K* disjoint components of the hypergraph whose size differences are controlled by the parameter *ϵ*. We observe a difference between the top-layer decomposition (where the tensor network is usually closed or contains few open edges) and the subsequent layers (where there are many open edges mostly connecting to other components). For this reason, we use the parameter combination (*K*, *ϵ*) for the top layer and $(2,\epsilon ^{\prime} )$ for subsequent layers. We then perform optimizations over the three parameters $(K,\epsilon ,\epsilon ^{\prime} )$ to obtain a satisfactory initial contraction tree. We use the covariance matrix adaptation evolution strategy algorithm^43^ for parameter optimization and the KaHyPar package for hypergraph decomposition^44^. The cutoff size for the hypergraph decomposition is set to 25; contraction trees on hypergraphs with fewer nodes are constructed greedily using built-in functionalities in the opt_einsum package^45^.

#### Index slicing and local optimization

After finding the initial contraction order, one way of selecting the indices to slice over is by greedily picking the index that decreases the space complexity the most or introduces the least time complexity overhead. In this work we interleave the greedy approach with a series of local reordering of the contraction tree that ensures a more robust slicing. In particular, we apply the following heuristics:

- The first one is a general local optimization method: take a connected subgraph of a contraction tree, which represents a series of contraction steps, with multiple intermediate outcomes as the input and a single output. Such a series of contraction steps represents a tensor network contraction of its own and can be optimized by reconfiguring the internal contraction tree connections. If the subgraph chosen is small enough, the optimal configuration can be found with a brute-force approach. Repeatedly choosing small connected subgraphs of the contraction tree and optimizing over them could greatly reduce the overall contraction cost. We focus on subgraphs with many high-cost intermediate steps to accelerate this process, which hopefully reduces the contraction cost by the maximum. In our experiments, we take subgraphs of size up to 14 to perform local optimizations on.
- The second one is more specifically designed for index slicing. In a contraction tree, the nodes in which a particular index appear form a subtree. The overhead induced by slicing a particular index is determined by the total cost of the corresponding subtree, which in turn depends almost entirely on the overlap of the subtree with the highest-cost nodes. The more high-cost nodes in a contraction tree involving a particular index, the less overhead is incurred while slicing this particular index. One can therefore slightly tweak the contraction tree by commuting different high-cost contraction steps to maximize the utility of a single index. This increases the overall unsliced cost (assuming that the original contraction tree is locally optimal), but at the same time reduces the slicing overhead via increasing the utility of the particular index. Enumerating over several promising index candidates helps find a good one, especially when an obvious choice is absent.

#### Runtime modification of the contraction scheme

When executing sequential pairwise contraction on a GPU, we apply the following runtime-specific modifications on the obtained contraction schemes. These modifications do not alter the theoretical contraction cost by much, but usually enable much more efficient execution.

- Most nodes in the contraction tree represent very small portions of the overall time complexity; however, they involve many small tensors, transmission of which to the GPU would incur considerable overhead. This motivates us to precompute these small steps on a CPU before executing the slicing and only deploy the heavy computational steps of each individual task on the GPU. The partial results for the low-cost steps are shared by all subtasks and only need to be computed once. In practice, this considerably reduces the communication cost between the GPU and the CPU and helps save a small portion of the computational cost. We regard any intermediate step resulting in an intermediate tensor of rank 23 (before slicing) as a low-cost step, and execute these steps before slicing.
- After the precomputation getting rid of repeated low-cost steps, the computation performed on the GPU is typically a sequential absorption of small tensors into one large tensor, or two large tensors merged together near the end. In either case, a contraction tree with locally optimal contraction costs typically suffers from a large skewness in dimensions during matrix multiplication. On Nvidia Tesla V100 GPUs, matrix multiplication with dimensions *M* × *N* and *N* × *K* is much more efficient when the dimensions *M*, *N* and *K* are all multiples of 32; however, a typical small tensor is often shaped 4 × 4, 8 × 8 or 16 × 16. To overcome this, we slightly tweak the contraction order in the following way: whenever a large tensor is to be contracted with some small tensors consecutively, we instead contract the smaller tensors first and contract this intermediate result with the large tensor, thereby ensuring that inefficiency by skewness does not occur whenever the large tensor is involved in the contraction. This increases the runtime contraction cost, but decreases the actual running time by making use of the efficient kernel functions of the Nvidia Tesla V100. This is a somewhat ad hoc solution to the low GPU efficiency induced by small tensor dimensions; we hope that more systematic approaches can be explored to increase the GPU efficiency.

### Sycamore random circuits

The Sycamore random quantum circuits used to benchmark ACQDP are introduced in Arute et al.^2^ and are available from the public Dryad repository^46^. Each Sycamore random circuit is parameterized with a single parameter *m*, has 53 qubits arranged in a diagonal square grid pattern reflecting the qubit layout of the Sycamore quantum processor, and is generated randomly from some simple rules. Namely, a Sycamore random circuit is composed of *m* cycles, each consisting of a single-qubit gate layer and a two-qubit gate layer, and concludes with an extra single-qubit gate layer preceding measurement in the computational basis. In the first single-qubit gate layer, single-qubit gates are chosen for each individual qubit independently and uniformly at random from $\{\sqrt{X},\sqrt{Y},\sqrt{W}\}$, where

$$\sqrt{X}=\frac{1}{\sqrt{2}}\left[\begin{array}{ll}1&-i\\ -i&1\end{array}\right],\ \sqrt{Y}=\frac{1}{\sqrt{2}}\left[\begin{array}{ll}1&-1\\ 1&1\end{array}\right],\ \sqrt{W}=\frac{1}{\sqrt{2}}\left[\begin{array}{ll}1&-\sqrt{i}\\ \sqrt{-i}&1\end{array}\right].$$

In each successive single-qubit gate layer, single-qubit gates are chosen for each individual qubit uniformly at random from the subset of $\{\sqrt{X},\sqrt{Y},\sqrt{W}\}$ that excludes the single-qubit gate applied in the previous cycle. In each two-qubit gate layer, two-qubit gates are applied to about one-quarter of all pairs of adjacent qubit in the qubit layout, in a regular pattern, such that at most one two-qubit gate is applied to each qubit. There are four different patterns, labeled A, B, C and D in ref. ^2^, and the eight-cycle pattern A, B, C, D, C, D, A, B is repeated over all the two-qubit layers. Two-qubit gates are decomposed into four *Z*-rotations determined by the cycle index and

$${{{\mathrm{fSim}}}}(\theta ,\phi )=\left[\begin{array}{llll}1&0&0&0\\ 0&\cos (\theta )&-i\sin (\theta )&0\\ 0&-i\sin (\theta )&\cos (\theta )&0\\ 0&0&0&{e}^{-i\phi }\end{array}\right],$$

where the parameters *θ* and *ϕ* are determined by the qubit pairing.

#### The random circuit sampling task

A quantum circuit *U* naturally defines a distribution ${{{{\mathcal{D}}}}}_{U}$ over bitstrings when all qubits are measured under the computational basis after executing the circuit on the all-zero state: ${{{{\mathcal{D}}}}}_{U}(x):= | \langle x| U| 0\rangle {| }^{2}$. Ideally, a quantum device executing *U* would sample from the distribution ${{{{\mathcal{D}}}}}_{U}$ exactly, but in practice many sources of hardware error causes the actual distribution to deviate from the ideal one. The linear XEB was used to measure the closeness of the output distribution to the ideal distribution^2^. It is defined as 2^*n*^〈*p*_*I*_(*x*)〉 − 1, where *n* is the number of qubits, *p*_*I*_(*x*) is the probability of *x* in the ideal distribution, and the expectation is taken over the output distribution. The XEB is 0 when the output distribution is uniform, and is 1 when the output distribution is ideal following the Porter–Thomas statistics. It was argued from numerical evidence that the aforementioned random quantum circuits had achieved an XEB of approximately 0.2%; however, simulating these circuits was estimated to be infeasible and thus this could not be directly verified.

Meanwhile, in this paper we require the classical simulation algorithm to satisfy a stronger criterion of approximate sampling, namely (*ϵ*, 0.2%)-unbiased noise approximate sampling, where *ϵ* is negligible (a rough estimation shows that *ϵ* < 6.4 × 10^−31^ in our algorithm). The definition of unbiased noise approximate sampling is as follows:

##### Definition 1 (ϵ, F)-UNA sampling

For a quantum circuit *U* as a unitary on *n* qubits, the task of *F*-UNA sampling is to generate independent and identically distributed samples from the distribution

$${{{{\mathcal{D}}}}}_{U}^{(F)}:= F\times {{{{\mathcal{D}}}}}_{U}+(1-F)\times {{{{\mathcal{U}}}}}_{n},$$

where ${{{{\mathcal{U}}}}}_{n}$ denotes the uniform distribution over {0, 1}^*n*^. Moreover, (*ϵ*, *F*)-UNA sampling generates independent and identically distributed samples from a distribution *ϵ* close to the distribution ${{{{\mathcal{D}}}}}_{U}^{(F)}$ under total variational distance.

We will discuss more about why this stronger criterion is used in Supplementary Section 3C. Meanwhile, we will note that if we can achieve (*ϵ*, 1)-UNA sampling in average time *T*, then there is a trivial method to achieve (*ϵF*, *F*)-UNA sampling in average time *FT* by yielding a genuine sample with probability *F* and a uniformly random bitstring otherwise. We adapt this method in our expirments, generating near-perfect samples from ${{{{\mathcal{D}}}}}_{U}$ and multiplying the final running time estimate with a factor *F* = 0.2%.

#### Frugal rejection sampling

We adopt a previously proposed framework^2,6^ to reduce (near-perfect) sampling from ${{{{\mathcal{D}}}}}_{U}$ into computation of probability amplitudes of individual or small batches of bitstrings. This framework assumes that the output distribution of a random quantum circuit is a randomly permuted Porter–Thomas distribution. This assumption implies that there is a small number *M* (*M* ≈ 10 for 53-qubit circuits) for which bitstrings *x* with probability *p*_*I*_(*x*) > *M*/*N* (where *N* is the number of all possible bitstrings; *N* = 2^53^ in this case) do not contribute much to the overall distribution, which naturally gives rise to a frugal rejection sampling algorithm that on average only needs to compute *M* individual probability amplitudes to generate one sample from ${{{{\mathcal{D}}}}}_{U}$.

The overhead of frugal rejection sampling can be further decreased by computing a small batch of amplitudes for related bitstrings at a time, which for tensor network-based methods can be done with almost no extra cost compared to computing a single amplitude. We note that we cannot generate multiple samples from a single batch because that will introduce unwanted correlation between samples, violating the independent and identically distributed requirement for UNA sampling; however, if the first randomly chosen bitstring in a batch is rejected, then we can try other bitstrings in the same batch until one of them is accepted. With a batch of 2^6^ = 64 bitstrings, the probability that one of them will be accepted is close to 1, thus lowering the overhead of frugal rejection from about 10× to 1×. This may introduce some further deviation from the ideal distribution ${{{{\mathcal{D}}}}}_{U}$, but the error is negligible assuming that the correlation between amplitudes in the same batch is negligible.

### QAOA for graph isomorphism discovery

The QAOA was first developed by Farhi, Goldstone and Gutman^47^ to solve combinatorial optimization problems. For a combinatorial optimization problem of the form $C:{\{0,1\}}^{n}\to {\mathbb{R}}$, which can be decomposed as a sum of local clauses $C=\mathop{\sum }\nolimits_{i = 1}^{m}{C}_{i}$ each acting only on a small number of bits, QAOA works by regarding the objective function *C* as a local Hamiltonian $\hat{C}={\sum }_{x}f(x)\left|x\right\rangle \left\langle x\right|=\mathop{\sum }\nolimits_{j = 1}^{m}{\hat{C}}_{j}$, and taking the ansatz that the state

$$\left|\overrightarrow{\gamma },\overrightarrow{\beta }\right\rangle ={e}^{-i{\beta }_{p}\hat{B}}{e}^{-i{\gamma }_{p}\hat{C}}\cdots {e}^{-i{\beta }_{1}\hat{B}}{e}^{-i{\gamma }_{1}\hat{C}}{(\left|+\right\rangle )}^{\otimes n}$$

defined by the mixing operator $\hat{B}=\mathop{\sum }\nolimits_{i = 1}^{n}{X}_{i}$ and the angle sequences $\overrightarrow{\gamma },\overrightarrow{\beta }\in {{\mathbb{R}}}^{p}$ approaches an eigenstate of $\hat{C}$ with either minimum or maximum eigenvalue with carefully chosen parameters $\overrightarrow{\gamma },\overrightarrow{\beta }$, even with a small QAOA depth *p*. As both $\hat{B}$ and $\hat{C}$ are sums of commuting local terms, the state $\left|\overrightarrow{\gamma },\overrightarrow{\beta }\right\rangle$ can be readily prepared using a quantum circuit.

The QAOA energy function with *p* layers is defined as

$${F}_{p}(\overrightarrow{\gamma },\overrightarrow{\beta }):= \langle \overrightarrow{\gamma },\overrightarrow{\beta }| \hat{C}| \overrightarrow{\gamma },\overrightarrow{\beta }\rangle ,$$

that is, the expectation value of the objective function *C*(*Z*) where the random string *Z* comes from measuring the quantum state $\left|\overrightarrow{\gamma },\overrightarrow{\beta }\right\rangle$ under the computational basis.

In order to use QAOA for graph isomorphism discovery, consider the Max-cut problem on a graph *G* = (*V*, *E*), with the simple objective function *C*(*x*) = ∑_(*u*, *v*)∈*E*_∣*x*_*u*_ − *x*_*v*_∣, where *x* ∈ {0, 1}^∣*V*∣^. Obviously, the QAOA energy function ${F}_{p}(\overrightarrow{\gamma },\overrightarrow{\beta })$ for the Max-cut problem does not depend on the ordering of vertices in *V* but only the structure of *G*. Two isomorphic graphs will therefore always give the same value for ${F}_{p}(\overrightarrow{\gamma },\overrightarrow{\beta })$, no matter how $\overrightarrow{\gamma }$ and $\overrightarrow{\beta }$ are chosen. On the other hand, it is conjectured that for two non-isomorphic graphs, for sufficiently large *p*, the values of ${F}_{p}(\overrightarrow{\gamma },\overrightarrow{\beta })$ are different with probability 1 for $\overrightarrow{\gamma },\overrightarrow{\beta }$ uniformly chosen from [0, 2*π*]^2p^ (ref. ^31^); thus, evaluating ${F}_{p}(\overrightarrow{\gamma },\overrightarrow{\beta })$ for two graphs *G*_1_ and *G*_2_ with randomly chosen $\overrightarrow{\gamma },\overrightarrow{\beta }$ can either reveal that *G*_1_ and *G*_2_ are non-isomorphic, or give a strong evidence that they are isomorphic. The larger *p* is, the stronger such evidence will be.

As the QAOA energy function can be written as a sum of energy values of all clauses, the above proposal is essentially a way to use the QAOA to characterize local neighborhoods of vertices in a graph. This locality also translates to ease of computation with tensor network-based methods. In order to compute the energy value of a clause, the tensor network to evaluate corresponds to only part of the QAOA circuit, namely the lightcone of that clause (see Supplementary Section 4C).

### Surface-17

Surface-17 (ref. ^35^) is a surface code involving 17 qubits, including nine data qubits, four X-ancilla qubits for *X* stabilizer measurements, and four Z-ancilla qubits for *Z* stabilizer measurements. The nine data qubits are arranged in a 3 × 3 grid, and the ancilla qubits all lie on another square lattice diagonally displaced from the data qubit lattice, such that each ancilla qubit is diagonally adjacent to either four data qubits, or two data qubits on the border of the 3 × 3 grid. During normal operation of the surface code, two-qubit gates are applied only between adjacent pairs of one data qubit and one ancilla qubit (not two data qubits nor two ancilla qubits). See Supplementary Fig. 7 for a diagram of the qubit layout.

#### Error model

The error model we use is based on the one in O’Brien et al.^35^, which includes idling, gate-specific and measurement errors. We choose not to include the gate-specific error of CZ gates—modelled as a quasi-static flux noise—as its quasi-static coherent nature enables various techniques to compensate for it. We do introduce a model for cross-talk error caused by stray 2-qubit ZZ interactions^48^. See Supplementary Section 5B^49^.

#### Logical memory experiment

In this paper, we study only how well the surface code preserves the value of a logical qubit (as opposed to how to initialize, apply a gate to, or measure the logical qubit). To detect and correct qubit errors that may happen even while idling, stabilizer measurements (also known as error syndrome extraction) need to be constantly performed. Our syndrome extraction circuits are based on the ones in O’Brien et al. ^35^, containing only *R*_*y*_( ± *π*/2) gates, CZ gates, and computational basis measurements. We also study variants of the syndrome extraction circuit where different stabilizers are measured by adding or removing some *R*_*y*_(±*π*/2) gates.

We consider an idealized experiment that ignores errors during initialization or the final measurement. Starting from any single-qubit state, we first encode it into Surface-17 with an ideal (noiseless) encoding circuit, then perform *k* rounds of noisy syndrome extraction plus 1 round of noiseless syndrome extraction (which we sometimes write simply as ‘*k* + 1 rounds of syndrome extraction’). The final round of noiseless syndrome extraction projects the physical state back to the code space, and allows us to map the final state back to a single-qubit logical state with a Pauli correction indicated by the *optimal decoder*. This entire process can be described by a well-defined logical channel *C* on the single logical qubit, which contains information on the kinds and magnitudes of all logical errors incurred by this process.

#### PTMs

We describe a single-qubit quantum channel as a PTM, a 4 × 4 real matrix indicating how the channel modifies the expectation values of Pauli operators. The PTM for a channel ${{{\mathcal{C}}}}$ is defined as

$$P{({{{\mathcal{C}}}})}_{ij}=\frac{1}{2}{{{\rm{Tr}}}}[{\sigma }_{i}{{{\mathcal{C}}}}({\sigma }_{j})],$$

where *σ*_0_, *σ*_1_, *σ*_2_, *σ*_3_ = *I*, *X*, *Y*, *Z*, respectively. Note that the first row of any PTM corresponding to a trace preserving map is always (1, 0, 0, 0), since a physical quantum channel should not change the expectation value of *I*, regardless of the expectation values of *X*, *Y*, and *Z*.

#### Optimal decoder

During normal operation of a stabilizer code, any errors detected are usually not corrected with physical gates. Instead, conceptually, virtual Pauli gates are applied to some of the code qubits, which is implemented by adjusting the results of stabilizer measurements thereafter on those qubits. Any Pauli gates on any number of code qubits can be implemented this way as long as the only operations applied on the code qubits are Clifford gates and stabilizer measurements.

Accordingly, our decoder tries to correct errors using only Pauli gates on code qubits, depending on the error syndromes measured. It is implemented in two steps: first, based on only the final round of noiseless error syndromes, a trivial decoder uses any number of Pauli gates to map the code qubits back into the code space. Second, based on all (including noisy and noiseless) error syndromes, a logical Pauli gate, one of *I*, *X*, *Y*, and *Z*, is applied to the logical qubit in order to maximize the fidelity of the logical channel. For the circuit sizes considered in this paper (no more than 2 + 1 rounds of syndrome extraction), tensor network-based simulations enables us to compute the optimal decoder exactly. See Supplementary Section 5D.

### Supplementary information

Supplementary Information Supplementary Figs. 1–8, Discussion and Supplementary Tables 1 and 2.

### Source data

Fig. 2 Source data for Fig. 2.

Fig. 3 Source data for Fig. 3.

Fig. 5 Source data for Fig. 5.

## Data Availability

All data^51^ used to create the figures in the main texts as well as in the Supplementary Information can be found at 10.5061/dryad.nk98sf7t8. Contraction orders were derived using the order-finding scheme in the ACQDP package. Detailed information about our cluster architecture and order-finding parameters can be found in Supplementary Section 2. Source data are provided with this paper.

## Code availability

ACQDP is publicly available at https://github.com/alibaba/acqdp. The specific version of the ACQDP package and the scripts^50^ that reproduces all of the results reported in this paper can be found at 10.24433/CO.4349832.v3.

## References

[1] Preskill, J. Quantum computing and the entanglement frontier. Preprint at https://arxiv.org/abs/1203.5813 (2012).

[2] Arute F (2019). Quantum supremacy using a programmable superconducting processor. Nature.

[3] Zhong, H.-S. et al. Quantum computational advantage using photons. *Science* **370**, 1460–1463 (2020). 10.1126/science.abe8770 33273064

[4] Preskill J (2018). Quantum computing in the NISQ era and beyond. Quantum.

[5] Steiger DS, Häner T, Troyer M (2018). ProjectQ: an open source software framework for quantum computing. Quantum.

[6] Boixo S (2018). Characterizing quantum supremacy in near-term devices. Nat. Phys..

[7] Pednault, E. et al. Breaking the 49-qubit barrier in the simulation of quantum circuits. Preprint at https://arxiv.org/abs/1710.05867 (2017).

[8] Biamonte JD, Morton J, Turner J (2015). Tensor network contractions for #SAT. J. Stat. Phys..

[9] Huang C, Newman M, Szegedy M (2020). Explicit lower bounds on strong quantum simulation. IEEE Trans. Inf. Theory.

[10] White SR (1992). Density matrix formulation for quantum renormalization groups. Phys. Rev. Lett..

[11] Vidal G (2003). Efficient classical simulation of slightly entangled quantum computations. Phys. Rev. Lett..

[12] Vidal G (2007). Classical simulation of infinite-size quantum lattice systems in one spatial dimension. Phys. Rev. Lett..

[13] Schollwöck U (2005). The density-matrix renormalization group. Rev. Modern Phys..

[14] Bravyi S, Suchara M, Vargo A (2014). Efficient algorithms for maximum likelihood decoding in the surface code. Phys. Rev. A.

[15] Ferris AJ, Poulin D (2014). Tensor networks and quantum error correction. Phys. Rev. Lett..

[16] Chubb CT, Flammia ST (2021). Statistical mechanical models for quantum codes with correlated noise. Ann. Henri Poincaré D.

[17] Darmawan AS, Poulin D (2018). Linear-time general decoding algorithm for the surface code. Phys. Rev. E.

[18] Dudek, J. M. and Vardi, M. Y. Parallel weighted model counting with tensor networks. Preprint at https://arxiv.org/abs/2006.15512 (2020).

[19] Schutski R, Khakhulin T, Oseledets I, Kolmakov D (2020). Simple heuristics for efficient parallel tensor contraction and quantum circuit simulation. Phys. Rev. A.

[20] Lykov, D., Schutski, R., Galda, A., Vinokur, V. & Alexeev, Y. Tensor network quantum simulator with step-dependent parallelization. Preprint at https://arxiv.org/abs/2012.02430 (2020).

[21] Villalonga B (2019). A flexible high-performance simulator for verifying and benchmarking quantum circuits implemented on real hardware. npj Quantum Inf..

[22] Orús R (2014). A practical introduction to tensor networks: matrix product states and projected entangled pair states. Annals Phys..

[23] Vidal G (2008). Class of quantum many-body states that can be efficiently simulated. Phys. Rev. Lett..

[24] Han Z-Y, Wang J, Fan H, Wang L, Zhang P (2018). Unsupervised generative modeling using matrix product states. Phys. Rev. X.

[25] Gao X, Zhang Z-Y, Duan L-M (2018). A quantum machine learning algorithm based on generative models. Sci. Adv..

[26] Markov IL, Shi Y (2008). Simulating quantum computation by contracting tensor networks. SIAM J. Comput..

[27] Boixo, S., Isakov, S. V., Smelyanskiy, V. N. & Neven, H. Simulation of low-depth quantum circuits as complex undirected graphical models. Preprint at https://arxiv.org/abs/1712.05384 (2017).

[28] Gray J, Kourtis S (2021). Hyper-optimized tensor network contraction. Quantum.

[29] Schutski R, Lykov D, Oseledets I (2020). Adaptive algorithm for quantum circuit simulation. Physical Review A.

[30] Pan, F. & Zhang, P. Simulating the sycamore quantum supremacy circuits. Preprint at https://arxiv.org/abs/2103.03074 (2021).

[31] Szegedy, M. What do QAOA energies reveal about graphs? Preprint at https://arxiv.org/abs/1912.12277 (2019).

[32] Wang H, Wu J, Yang X, Yi X (2015). A graph isomorphism algorithm using signatures computed via quantum walk search model. J. Phys. A.

[33] Emms D, Severini S, Wilson RC, Hancock ER (2009). Coined quantum walks lift the cospectrality of graphs and trees. Pattern Recognit..

[34] Mahasinghe A, Izaac JA, Wang JB, Wijerathna JK (2015). Phase-modified CTQW unable to distinguish strongly regular graphs efficiently. J. Phys. A.

[35] O’Brien TE, Tarasinski B, DiCarlo L (2017). Density-matrix simulation of small surface codes under current and projected experimental noise. npj Quantum Inf..

[36] Trout CJ (2018). Simulating the performance of a distance-3 surface code in a linear ion trap. New J. Phys..

[37] Ataides JPB, Tuckett DK, Bartlett SD, Flammia ST, Brown BJ (2021). The XZZX surface code. Nat. Commun..

[38] Zhou Y, Stoudenmire EM, Waintal X (2020). What limits the simulation of quantum computers?. Phys. Rev. X.

[39] Verstraete F, Murg V, Cirac JI (2008). Matrix product states, projected entangled pair states, and variational renormalization group methods for quantum spin systems. Adv. Phys..

[40] Pirvu B, Murg V, Cirac JI, Verstraete F (2010). Matrix product operator representations. New J. Phys..

[41] Verstraete F, Garcia-Ripoll JJ, Cirac JI (2004). Matrix product density operators: simulation of finite-temperature and dissipative systems. Physical review letters.

[42] Noh K, Jiang L, Fefferman B (2020). Efficient classical simulation of noisy random quantum circuits in one dimension. Quantum.

[43] Hansen, N., Akimoto, Y. & Baudis, P. *CMA-ES/pycma on Github* (Zenodo, 2019); 10.5281/zenodo.2559634

[44] Schlag, S. *High-Quality Hypergraph Partitioning.* PhD thesis, Karlsruhe Institute of Technology (2020).

[45] Daniel G (2018). Opt_einsum—a python package for optimizing contraction order for einsum-like expressions. J. Open Source Software.

[46] Martinis, J. M. et al. *Quantum Supremacy Using a Programmable Superconducting Processor* (Dryad, 2021); 10.5061/dryad.k6t1rj8

[47] Farhi, E., Goldstone, J. & Gutmann, S. A quantum approximate optimization algorithm. Preprint at https://arxiv.org/abs/1411.4028 (2014).

[48] DiCarlo L (2009). Demonstration of two-qubit algorithms with a superconducting quantum processor. Nature.

[49] Huang, C. et al. *Efficient Parallelization of Tensor Network Contractions for Simulating Quantum Computation* (Dryad, 2021); 10.5061/dryad.nk98sf7t8 10.1038/s43588-021-00119-7 PMC10766539 38217127

[50] Huang, C., Zhang, F. & Chen, J. An open-source simulator-driven development tool for quantum computing. *Code Ocean*10.24433/CO.4349832.v2 (2021).

[51] Meringer M (1999). Fast generation of regular graphs and construction of cages. J. Graph Theory.

[52] Brouwer, A. E. *Paulus-Rozenfeld Graphs* https://www.win.tue.nl/~aeb/graphs/Paulus.html

