# Supplementary material for: Efficient parallelization of tensor network contraction for simulating quantum computation
Source: Nat Comput Sci. 2021 Sep 13;1(9):578–87. doi: 10.1038/s43588-021-00119-7 (PMC10766539; doi:10.1038/s43588-021-00119-7)
Supplement: Supplementary file 1 — Supplementary Figs. 1–8, Discussion and Supplementary Tables 1 and 2. [file 43588_2021_119_MOESM1_ESM.pdf]

nature portfolio

https://doi.org/10.1038/s43588-021-00119-7

Supplementary information

Efficient parallelization of tensor network contraction for simulating quantum computation

In the format provided by the
authors and unedited

Supplementary Information for: Efficient Parallelization of Tensor Network Contraction for Simulating Quantum Computation

Cupjin Huang,[1,*] Fang Zhang,[1,2,*] Michael Newman,[3] Xiaotong Ni,[4] Dawei Ding,[1] Junjie Cai,[5] Xun Gao,[1] Tenghui Wang,[4] Feng Wu,[4] Gengyan Zhang,[4] Hsiang-Sheng Ku,[4] Zhengxiong Tian,[6] Junyin Wu,[5] Haihong Xu,[6] Huanjun Yu,[6] Bo Yuan,[6] Mario Szegedy,[1] Yaoyun Shi,[1,†] Hui-Hai Zhao,[7] Chunqing Deng,[4] and Jianxin Chen[1,‡]

[1]*Alibaba Quantum Laboratory, Alibaba Group USA, Bellevue, WA 98004, USA*
[2]*Department of Electrical Engineering and Computer Science, University of Michigan, Ann Arbor, MI 48109, USA*
[3]*Departments of Physics and Electrical and Computer Engineering, Duke University, Durham, NC 27708, USA*
[4]*Alibaba Quantum Laboratory, Alibaba Group, Hangzhou, Zhejiang 310000, China*
[5]*Alibaba Cloud Intelligence, Alibaba Group USA, Bellevue, WA 98004, USA*
[6]*Alibaba Cloud Intelligence, Alibaba Group, Hangzhou, Zhejiang 310000, China*
[7]*Alibaba Quantum Laboratory, Alibaba Group, Beijing, Beijing 100000, China*

(Dated: July 29, 2021)

I. ALGORITHM AND HEURISTICS FOR TENSOR NETWORK CONTRACTION

This work's main contribution is an efficient framework for contracting tensor networks of size previously thought out of reach. Among many techniques applied, what greatly enlarged the scope of such contraction is an embarrassing parallelization by sub-dividing a tensor network contraction task into subtasks that can be executed simultaneously while introducing little overhead.

Tensor network is a generalization of several common definitions of "multiplication" in linear algebra. To motivate the basic idea of tensor networks, we take the simplest example of the matrix multiplication, where two matrices A, B are taken as input, and the dot product of A and B are defined as

$$C = A \cdot B \Leftrightarrow C_{ik} = \sum_j A_{ij} B_{jk}. \tag{1}$$

The product C is again a matrix, whose entries are defined by the entries of A and B as given in Equation (1). A tensor network generalizes the notion of matrix multiplication in the following ways:

- There can be an arbitrary number of input operands as opposed to exactly two.
- Each operand can be a *tensor* rather than a matrix, i.e. it can have an arbitrary number of indices as opposed to 2.
- The indices appearing in the input operands can be "connected" arbitrarily, as long as all the indices connected together have matching ranges. Such connected indices can either be summed over (i.e. contracted) or they can remain and serve as indices of the output, which is again a tensor.

In the rest of the subsection, we define tensors and tensor networks formally. For a positive integer $d \in \mathbb{Z}_+$, we use $[d]$ to indicate the set $\{0, \ldots, d-1\}$ for $d \in \mathbb{N}_+$. For a list of positive integers $\vec{d} = (d_1, \ldots, d_n)$, we denote $[\vec{d}]$ as the Cartesian product $[\vec{d}] := \times_{i=1}^n [d_i]$.

A. Tensors and tensor networks

a. Tensors In our context, a tensor is a multi-dimensional array of complex numbers $T \in \mathbb{C}^{\times_{i=1}^r d_i}$. The number of dimensions r is called the *rank* of the tensor, and the value of each dimension $\vec{d} = (d_1, \ldots, d_r)$ is called the *shape* of the tensor, where each entry is called the *bond dimension* associated with the corresponding index. For a specific index assignment $a = (a_1, \ldots, a_n) \in [\vec{d}]$, we use brackets to represent the indexing referring to an entry: $T[a] \in \mathbb{C}$.

---

*C.H. and F.Z. contributed equally to this work.
†Electronic address: y.shi@alibaba-inc.com
‡Electronic address: liangjian.cjx@alibaba-inc.com

b. Tensor networks A tensor network defines a multilinear function mapping some input tensors into an output tensor. A tensor network is associated with an attributed multi-hypergraph $H = (V, E)$, where

1. there is a mapping $d : E \to \mathbb{N}^+$ from each hyperedge to a positive integer, called the *bond dimension* of the hyperedge, and
2. for each vertex v, there is an ordering $o_v : [\deg(v)] \to \{e \in E | v \in e\}$ of the hyperedges incident to it, and
3. there is a subset of hyperedges E_o called *open edges*.

Each hyperedge connects among indices from potentially different vertices in the tensor network with the same bond dimension. These hyperedges are used to define a multilinear function via the Feynman path integral procedure. Each vertex v takes as the input a tensor with shape matching the hyperedge connections $T_v \in \mathbb{C}^{\times_{i=1}^{\deg(v)} d(o_v(i))}$. An assignment of all the hyperedges in the tensor network, i.e. an element $a \in [(d(e))_{e \in E}]$, fixes indices of all tensors in the tensor network, and is associated with the product of all the corresponding entries via a Feynman path:

$$F(a) := \prod_{v \in V} T_v[(a_{o_v(1)}, a_{o_v(2)}, \ldots, a_{o_v(\deg(v))})].$$

Given a tensor network $H = (V, E)$ and the input tensors $(T_v)_{v \in V}$, the *value* associated with the tensor network is a tensor $T_H \in \mathbb{C}^{\times_{e \in E_o} d(e)}$ so that the entry of the partial assignment b is the summation of all possible Feynman paths whose index assignments agree with b:

$$T_H[b] := \sum_{a \in [(d(e))_{e \in E}]; \forall e \in E_o, a_e = b_e} F(a).$$

The computational task of solving for the value of the multilinear function given the input tensor values is called *contraction* of the tensor network.

For the purpose of classically simulating qubit quantum circuits, it is usually sufficient to assume that all bond dimensions appearing are 2. main text Figure 1 a illustrates a small tensor network consisting of 4 tensors and 5 hyperedges, where each hyperedge is colored differently and corresponds to the index with the same color on the right.

B. Contraction trees and tensor network slicing

The Feynman path approach illustrated above is space-efficient. Still, it is hardly feasible in practice, as the time complexity of enumerating all Feynman paths grows exponentially with respect to the number of hyperedges. In fact, it is known that exactly contracting a tensor network is a computationally hard, #P-complete problem. In this subsection, we review two alternative strategies for tensor network contraction.

The first one, called *sequential pairwise contraction*, is a direct generalization of the state vector update method. At each step, two vertices in the tensor network are chosen. The corresponding tensors associated with the two nodes are multiplied together, similar to matrix multiplication. The corresponding two-node subgraph is then replaced by a single vertex preserving all the outgoing connections, with the updated tensor associated with it. This step is repeated until only one tensor is left in the tensor network, whose value equals the value of the tensor network. The time and space complexity of sequential pairwise contraction can be significantly reduced by choosing good contraction orders.

The second one, called *tensor network index slicing*, is a hybrid between the Feynman path integral and the sequential pairwise contraction. Unlike the Feynman path integral where *all* indices are enumerated over, only a carefully chosen subset of indices are enumerated. For each partial assignment on the small subset of indices, a smaller, often simpler tensor network can represent the corresponding partial sum of the Feynman paths. Evaluating each of the smaller tensor networks by sequential pairwise contraction and then summing up all the partial results give value of the full tensor network. This idea is further enhanced with more careful selections of indices to slice in order to reduce the parallelizing overhead.

a. Contraction trees Disregarding the exact order that pairwise contractions happen and focusing on the dependency between the steps, we formulate the idea of *sequential pairwise tensor contraction* into a computational graph called the *contraction tree*. The leaf nodes in a contraction tree represent the input tensors in the tensor network. Each internal node represents the intermediate step of contracting its two children, and the root node represents the final result. main text Figure 1 b depicts a contraction tree that corresponds to the tensor network in main text Figure 1 a.

The final result of the contraction does not depend on the specific contraction tree one chooses to pairwise contract the tensors, but only on the hypergraph structure and the specific values of the input tensors. In contrast, the time (space) complexity, defined as the summation (maximum) of the time (space) complexities of the stepwise contractions, does not depend on the specific values of the input tensors, rather just the time and space complexities for each intermediate step, which in turn only depend on

the rank of the intermediate tensors and their connections. This motivates us to ignore the values of the input tensors for now, and focus on the unattributed hypergraph when investigating the contraction trees.

Formally, a contraction tree $T = (V \cup V', E_T)$ associated with a tensor network $H = (V, E)$ is a rooted binary tree with root r, where each node in V is a leaf node and each node in V' has exactly two children. Each leaf node is associated with the hyperedges it is connected to in H, and each non-leaf node u with children A and B is associated with the set $E_u = E_{AB}$. Furthermore, for a non-leaf node u with children (A, B) we denote $E_u^* := E_{AB}^*$. It can be shown that each hyperedge is present in the minimum connected subgraph of T spanned by the leaf nodes adjacent to it, and the root node for open edges.

For an intermediate step contracting two nodes A and B, denote the two vertices as A, B, let E_{AB} be all of the edges that are either connected to A or B, and let $E_{AB}^* \subset E_{AB}$ be the edges in E_{AB} that are open or are connected to other vertices. The stepwise contraction enumerates over all Feynman paths associated with the hyperedges in E_{AB}, and sums up those that agrees on E_{AB}^*. The resulting time and space complexities for a step are then $2^{|E_{AB}|}$ and $2^{|E_{AB}^*|}$ respectively. The total *time complexity* associated with the contraction tree T is defined as the sum of time complexities for each step:

$$tc(T, H) := \sum_{u \in V'} 2^{|E_u|},$$

while the *contraction width* of a contraction tree T, which serves as an indicator of the space complexity, is defined as the maximum over all intermediate steps:

$$sc(T, H) := \max_{u \in V'} |E_u^*|.$$

Sequential pairwise contractions are generally considered the most efficient way to contract tensor networks with respect to total time complexity. However, it has several disadvantages. First, it is inherently sequential in the sense that a contraction tree must be executed bottom up; a little bit of parallelism can in principle be obtained from executing each layer in the contraction tree in parallel, but that does not provide significant speed-up. Second, sequential pairwise contractions have an inherent space complexity lower bound associated with the *contraction width* of the hypergraph. If a hypergraph is too big, it is possible that no sequential contraction orders can fit into a machine with limited memory. Also, finding an efficient contraction tree is an NP-hard combinatorial optimization problem, although approximate algorithms exist [1]. One then needs to apply heuristics to find good contraction trees in practice.

b. Slicing One of the main features of our work is using index slicing to greatly mitigate the inherent sequentiality and memory limits of the sequential pairwise contraction, without introducing a noticeable overhead in the total time complexity. Slicing begins by choosing a subset of hyperedges. Each assignment to those hyperedges induces a sub tensor network, whose hypergraph structure is identical to the original one but with the fixed hyperedges removed. The value of the full tensor network can then be obtained by enumerating over all possible assignments on the chosen hyperedges and then summing the values of the sub tensor networks together, see main text Figure 1 c.

Given the set of sliced hyperedges E_s of a hypergraph $H = (V, E)$, all of the subtasks, though differing in tensor values, are associated with the same sub hypergraph $H_s = (V, E \setminus E_s)$. The sub tensor networks can either be contracted via a pairwise contraction tree or sliced recursively. However, the latter can be reduced to the former without loss of generality by slicing over the union of all the chosen hyperedges.

A slicing-incorporated tensor network contraction scheme is then a pair (E_s, T) where E_s is the set of sliced hyperedges and T is the contraction tree on the sub hypergraph $H' = (V, E \setminus E_s)$. The time complexity and the contraction width of this contraction scheme are then

$$tc((E_s, T), H) := 2^{|E_s|} tc(T, H'),$$

$$sc((E_s, T), H) := sc(T, H').$$

A full complexity metric of a slicing-incorporated contraction scheme is characterized by a tuple: the *contraction cost* or the time complexity tc, the space complexity sc, and the number of subtasks indicated by the number of sliced indices. We would like to note that the space complexity of a contraction scheme is not necessarily the memory footprint for an actual implementation, since multiple large tensors could coexist at the same time. However, in most scenarios of interest there are only a small number of large tensors in any single timestep, hence the space complexity, defined to be the largest size of any intermediate tensor, closely captures the actual memory usage. On the other hand, the time complexity is defined to be the sum of the time complexities for each individual step rather than the biggest one. This is because the polynomial prefactor introduced by the number of steps could make a huge difference in the total time complexity, which can be up to 2 to 3 magnitudes for the sizes of problems of interest.

Real-world computational devices face much harder limits in space than in time, since it is relatively easy to run an algorithm for a longer time than to extend the physical memory of a device. Therefore, the goal of finding a good contraction scheme

can be formulated as minimizing the total time complexity subject to the constraint that the space complexity is bounded by a predetermined memory limit. For an Nvidia V100 with 16 GiB of memory, it is usually sufficient to slice the tensor network so that the contraction width is at most 29, assuming single precision complex numbers. We hereby adopt this convention, and only report the contraction cost and number of subtasks for the tensor network contraction tasks mentioned throughout the paper, assuming the space complexity is upper bounded by 2^{29}. Also, for sake of simplicity, the contraction cost reported throughout this work is the base-10 logarithm of the actual computational cost.

In general, index slicing is a method for parallelizing an existing contraction tree, and it inevitably brings overhead: $tc((E_s, T), H) \geq tc(T, H)$. Moreover, finding good indices to slice over is again a combinatorial optimization problem that is NP-hard. Here we develop intertwined framework of optimizing over both the contraction tree and the index slicing that helps find good slicing-incorporated contraction tree that significantly reduce the overhead of parallelization.

C. Optimization approach for finding good contraction scheme

Finding a good contraction scheme involves finding simultaneously a good contraction tree and indices to slice. Unfortunately, both of them are combinatorial optimization problems that are NP-hard. Multiple heuristics have been developed to find good contraction trees [1, 2] and index slicing. Here we propose a two-phase contraction-scheme finding framework, whose flowchart is presented in main text Figure 1 d. The idea is first to find a good unsliced contraction tree, and then to find indices to slice as well as to keep the contraction tree locally optimal.

a. Initial contraction tree finding Efficient tensor network contraction by finding low-cost contraction trees dates back to Markov and Shi [1], where it is shown that tensor network contraction can be done in time polynomial with respect to the time complexity associated with the optimal contraction tree, using a technique called *tree decomposition*. With highly efficient tree decomposition packages such as quickBB [3] and PACE [4], people have significantly expanded the scope of classically simulable tensor networks [5]. However, tree-decomposition-based methods fall short at resolving two issues. First, tree-decomposition-based approaches finds a low-cost contraction tree by minimizing the treewidth, i.e. the maximum time complexity of a single steps, thus fail to address the polynomial factor induced by the number of intermediate steps, which could be over a thousand for NISQ applications. Second, the tree-decomposition-based approaches only account for low total time complexity. Although this usually also leads to relatively small space complexity, it fails to address cases where space complexity becomes critical.

To remedy these shortcomings of tree-decomposition-based approaches, Gray and Kourtis [2] adopted the idea of hypergraph decomposition in order to minimize the overall time complexity. Hypergraph-decomposition-based method tries to construct contraction trees top-down, by dividing a tensor network into several components, for which contraction trees are found recursively in a divide-and-conquer manner. The contraction tree constructed will then first contract each component into a single tensor, and then merge the tensors together. The components are chosen such that the inter-component connections are minimized in some measure, which often results in a reduced time and space complexity on the top layer. The performance of hypergraph-decomposition-based approaches is further enhanced by introducing randomness and tunable parameters for optimization purposes.

A hypergraph decomposition algorithm takes a hypergraph and two parameters: the number of components to obtain K and the imbalance parameter ϵ which controls the size of the largest component from the decomposition. The algorithm then tries to find a K-partition of the hypergraph such that some objective function, typically the multi-partite cut, is minimized. In Gray and Kourtis [2], the authors did recursive multi-partition using the same parameter combination for all layers (K, ϵ), and applied an optimization scheme over the two parameters.

In our work, we apply a slight augmentation of the hypergraph-decomposition-based contraction tree construction, based on our observation of a typical contraction tree of tensor networks originated from quantum circuits with nearest neighbor connections. It is observed that a typical contraction tree has a stem-branch structure: All nodes in the contraction tree representing a high-cost matrix multiplication tend to form a relatively short path in the tree, while most of the intermediate steps are low cost, and form small clusters attached to the stem. This represents a typical scenario during tensor network contraction, in which a small cluster of tensor nodes are contracted together and absorbed into a big "stem" tensor node, resembling the update of a state vector. Depending on the position of the root with respect to the stem, such a contraction tree will often be of a Λ shape or a I shape. This inspires us to treat the top layer hypergraph decomposition differently from the lower level ones: For lower levels, we usually need an imbalanced bipartition to separate a branch from the stem, whereas in the top layer we usually need a more balanced multi-partition to separate the stem cluster(s), as well as some residual components that might be contracted together with the two principal stem components. For this reason, we use a parameter combination (K, ϵ) for the top-layer decomposition, and $(2, \epsilon')$ for subsequence layers. The optimization is then performed on the three parameters (K, ϵ, ϵ').

b. Robust index slicing After finding a good initial unsliced contraction tree, the task is then to select a few indices to slice over to enable parallelism and at the same time relieve the space constraint. One naïve approach to find indices to slice is by greedily choosing the index that minimizes the cost after slicing at each step. This method proved useful when the number of indices to slice is small, but might result in an unnecessarily large amount of indices sliced when the tensor network is too

complex. A recent work by Lykov et al. [6] further refined on the algorithm and introduced state-dependent slicing for enhanced performance.

In this work, we improve upon the previous greedy index slicing scheme, by interleaving it with a series of local reordering of the contraction tree that ensures a more robust slicing. The idea of local reordering is illustrated in Figure 1.

- The first one is a general local optimization method: Take a connected subgraph of a contraction tree, which represents a series of contraction steps, with multiple intermediate outcomes as the input and a single output. Such a series of contraction steps represent a tensor network contraction of its own, and can be optimized by reconfigure the internal contraction tree connections. If the subgraph is chosen small enough, then a brute-force approach can be applied to find the optimal internal connections. Repeatedly choosing small connected subgraphs of the contraction tree and optimizing over them could greatly reduce the overall contraction cost. To accelerate the local reconfigurations, we focus on subgraphs that overlaps heavily with the stem of the tree, which hopefully reduces the contraction cost by the most.

- The second one is more focused on index slicing. In a contraction tree, the nodes where a particular index appear forms a subtree. Since the stem takes up a dominant part of the cost in a contraction tree, the utility of an index, namely the overhead induced by slicing a particular index, is almost determined by the overlap of its corresponding subtree and the stem, which forms an interval on the stem. In some cases, there is an index whose subtree overlaps almost entirely with the stem, meaning that slicing it introduces a very small overhead. In other cases, none of the index subtrees overlap much with the stem. In this case, one can slightly tweak the contraction tree by commuting the outgoing branch of an index from the stem with its neighbor. This increasing the overall unsliced cost (assuming that the original contraction tree is locally optimal), but at the same time reduces the slicing overhead via increasing the overlap of the subtree with the stem. This helps find good indices to slice when an obvious choice is absent.

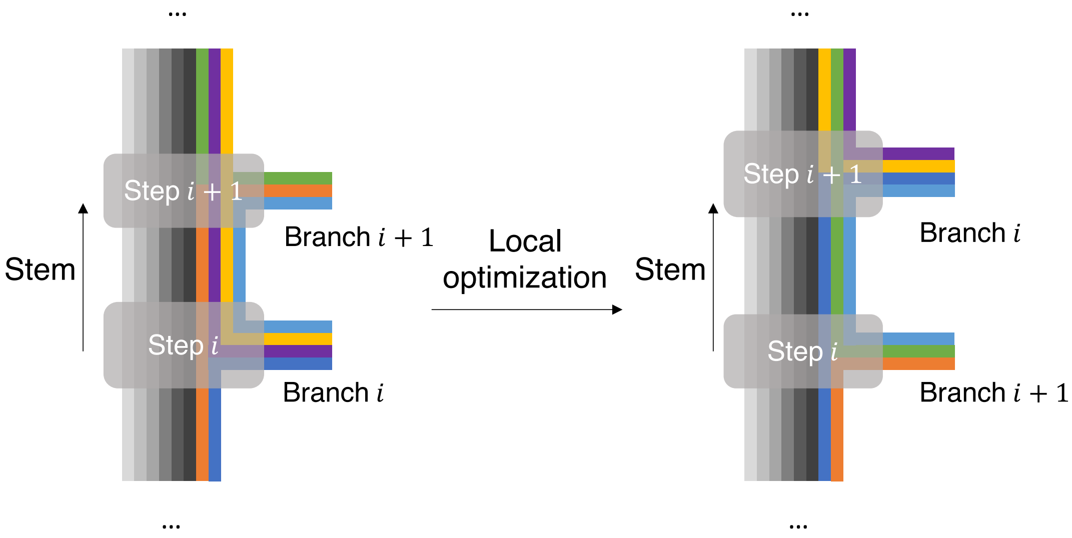

Supplementary Figure 1: Illustration of stem-based local optimization. By switching the branches i and $i+1$, both the space and time complexity of the contraction are reduced.

II. EXPERIMENTS

A. Cluster architecture

We use the Alibaba Cloud clusters to conduct numerical experiments. Though cluster architectures may suffer from higher latency in communication between nodes than a supercomputer, our tensor network-based computation requires little communication.

The cluster architecture consists of a single node as an agent to split the large tensor network contraction task into many smaller tensor network contraction subtasks. This step is called *preprocessing*. Then, the agent node uses the OSS (Object

Storage Service) as a data transmission hub to assign different subtasks to different computation nodes. When a computation node is finished with the assigned subtask, it will upload the result to the OSS, and the agent node will repeatedly query the OSS until all the subtask results add up to the desired amplitude. The reported *runtime* is the total elapsed time obtained on the agent node except for the preprocessing step. This is because we only need to do preprocessing once on a single node (the agent node), independent of the number of amplitudes we will calculate. Therefore, the preprocessing step is negligible in terms of both runtime and core-hours for sampling tasks that usually involve thousands of amplitudes and many computation nodes.

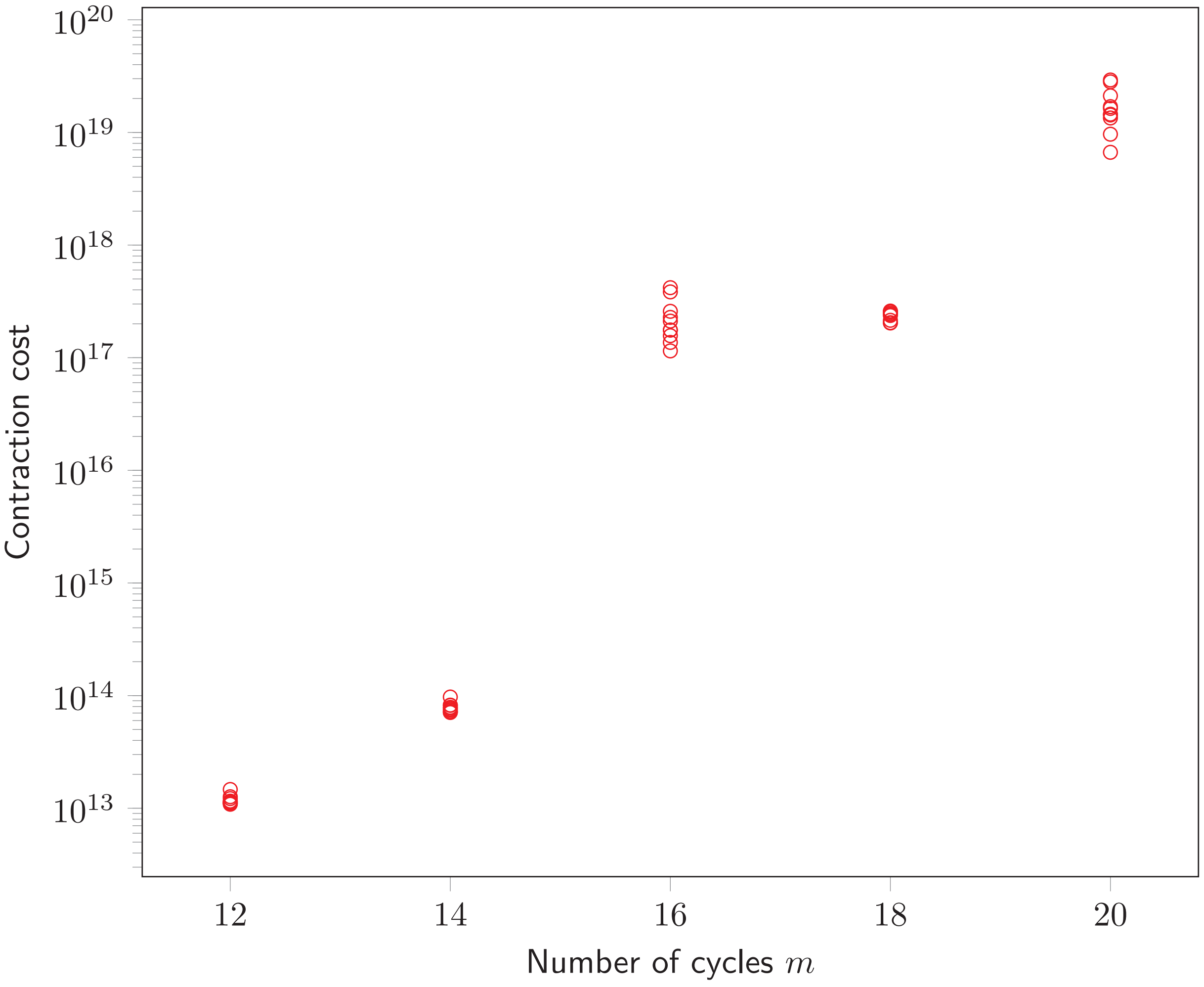

Supplementary Figure 2: For each length, we run preprocessing for the quantum circuits in Arute et al. [7] ten times. In main text Figure 3 we report the sliced and unsliced contraction cost we obtained over all ten runs in a box plot. Here, we summarize the sliced contraction cost for all runs for each depth with a scattered plot. Each point indicates the final contraction cost of one contraction order found in ten runs.

Simulating Bristlecone-70 circuits with ACQDP v0.1.0: A large-scale simulation of Bristlecone-70 circuits using double precision was conducted on an Alibaba Cloud cluster composed of 1449 Elastic Compute Service (ECS) instances, each with 88 Intel Xeon (Skylake) Platinum 8163 vCPU cores @ 2.5 GHz and 160 GiB of memory as computation nodes. We calculated $200,000$, $1,000$, 200, and 1 amplitudes of Bristlecone-70 circuits with depth $1+28+1$, $1+32+1$, $1+36+1$, and $1+40+1$, respectively. The last two were the first successful simulations of instances at those depths. For the sake of comparison, we also created 4 ECS instances with 2, 4, 8, and 16 vCPU cores, respectively, as part of a "virtual" cluster of $127,512$ vCPU cores. Those ECS instances supported Memory-to-CPU-core ratios of 2. For a large tensor network contraction task that has been split into K subtasks that each fit into 2 GiB of memory, we ran $\frac{K}{127,512}$ subtasks on each vCPU core. We use the largest execution time on those ECS instances to extrapolate the execution time of the whole tensor network contraction task on the large cluster. Comparing the results, we concluded that scheduling and communication costs are negligible. Detailed benchmarking results can be found in Table 1. Based on the 200,000 amplitudes we calculated for Bristlecone-70 circuits of depth 1 + 28 + 1, we plot the distribution of N_p in Figure 3, which closely matches the Porter-Thomas form.

Extrapolated execution time on a cluster with 127, 512 vCPU cores			
Circuit	Amplitudes	Subtasks p.a.	Seconds p.a.
Bristlecone-70×(1 + 28 + 1)	200,000	256	0.03
Bristlecone-70×(1 + 32 + 1)	10,000	1024	0.36
Bristlecone-70×(1 + 36 + 1)	100	65536	4.56
Bristlecone-70×(1 + 40 + 1)	1	4194304	480.17

Actual execution time on a cluster with 127, 512 vCPU cores			
Circuit	Amplitudes	Subtasks p.a.	Seconds p.a.
Bristlecone-70×(1 + 28 + 1)	200,000	256	0.04
Bristlecone-70×(1 + 32 + 1)	1,000	1024	0.43
Bristlecone-70×(1 + 36 + 1)	200	65536	5.75
Bristlecone-70×(1 + 40 + 1)	1	4194304	580.7

Supplementary Table 1: Extrapolated and actual execution times for the simulation of random circuit sampling on Bristlecone by using ACQDP version 0.1.0 on an Alibaba Cloud cluster of 127, 512 vCPU cores.

Simulating Sycamore random circuits with ACQDP v0.1.1: We use a "virtual" Alibaba Cloud cluster, with resources comparable to the Summit supercomputer, to estimate the cost of the proposed superiority task, namely sampling one instance of a given quantum circuit a million times with a given XEB fidelity. Our "virtual" cluster consists of 27, 648 *ecs.gn6v-c8g1.2xlarge* instances acting as computational nodes and an *ecs.g6.6xlarge* instance acting as an agent node to handle preprocessing and scheduling. Since our previous large-scale simulation demonstrated that scheduling and communication times are negligible, we can use the execution time on one computational node to extrapolate the execution time on the cluster. The agent node, an *ecs.g6.6xlarge* instance, has 24 Intel Xeon (Skylake) Platinum 8163 vCPU cores @ 2.5 GHz and 96 GiB of memory. A computation node, an *ecs.gn6v-c8g1.2xlarge* instance, has 8 Intel Xeon (Skylake) Platinum 8163 vCPU cores @ 2.5 GHz and 32 GiB of memory, as well as an Nvidia Tesla V100 SMX2 GPU with 16 GiB of RAM. We use single precision. In order to make best use of the GPU, we use the JAX library to just-in-time compile our contraction scheme. Since all subtasks for a sampling task use the same contraction scheme, the compilation only needs to be done once on each computation node, and the overall time spent is negligible. Detailed benchmarking results can be found in main text Figure 3.

B. Comparison with other simulators

We performed extensive comparisons with other tensor network-based simulators, including qFlex [8, 9] and Cotengra [2].

In Arute et al. [9], benchmarked runtimes and FLOPs of calculating batches of 64 amplitudes on the Summit supercomputer at Oak Ridge National Laboratory were reported. We perform our runtime estimation by using exactly the same number of Elastic Compute Service nodes (27648) with exactly the same graphics card model (Nvidia Tesla V100 SXM2 16 GB) as the Summit supercomputer, but on Alibaba Cloud. We tested our framework across different Alibaba Cloud regions, which could affect the performance due to lack of low-latency interconnects across regions. However, because our algorithm introduces very little communication, the communication overhead is negligible. Since both are targeting the same sampling task by assuming Summit-comparable computational resources, we use the numbers reported in Arute et al. [9].

In Gray and Kourtis [2], benchmarked times and other information for computing single amplitudes of random circuits with single precision using Nvidia Quadro P2000s were reported. In order to conduct a fair comparison, we re-ran the latest GitHub version of Cotengra [37] to calculate single amplitudes with perfect fidelity on the same Elastic Compute Service node with the same graphics card (Nvidia Tesla V100 SXM2 16 GB) that we used to benchmark ACQDP. In ACQDP, the preprocessing step is controlled by the number of iterations in the CMA-ES algorithm and the number of iterations of local optimization. Consequently, there is no way to precisely control the preprocessing time. However, by using the iteration parameters mentioned above, it usually takes from one hour to several hours, depending on number of cycles. To avoid underestimating Cotengra and to better understand its ultimate capability, we allow the path optimizer in Cotengra to search for 10 hours, which should always be large enough to compare with the pre-processing time used in the ACQDP benchmark. We also discussed with the developer of Cotengra about better strategies of using Cotengra. As they suggested, we execute 5 optimizers for 2 hours each, with more exhaustive slicing after each, then choosing the best and time a run. The reported number for Cotengra in this manuscript is the best from 5 runs.

The simulation proposal in Pednault et al. [10] leverages secondary storage to assist main memory when the quantum state is too large to fit. Any proposal that needs to store the entire state vector is limited by the storage space available. The Summit supercomputer has 250 PiB of secondary storage, while a 54-qubit state vector stored in single precision takes 128 PiB to store. The space requirement doubles with each additional qubit, and so this proposal would already have trouble scaling to 55-qubit circuits, while 56-qubit circuits would be out of reach.

The time estimate in Pednault et al. [10] is based on the runtimes reported in Häner and Steiger [11], in which 0.5 PiB of

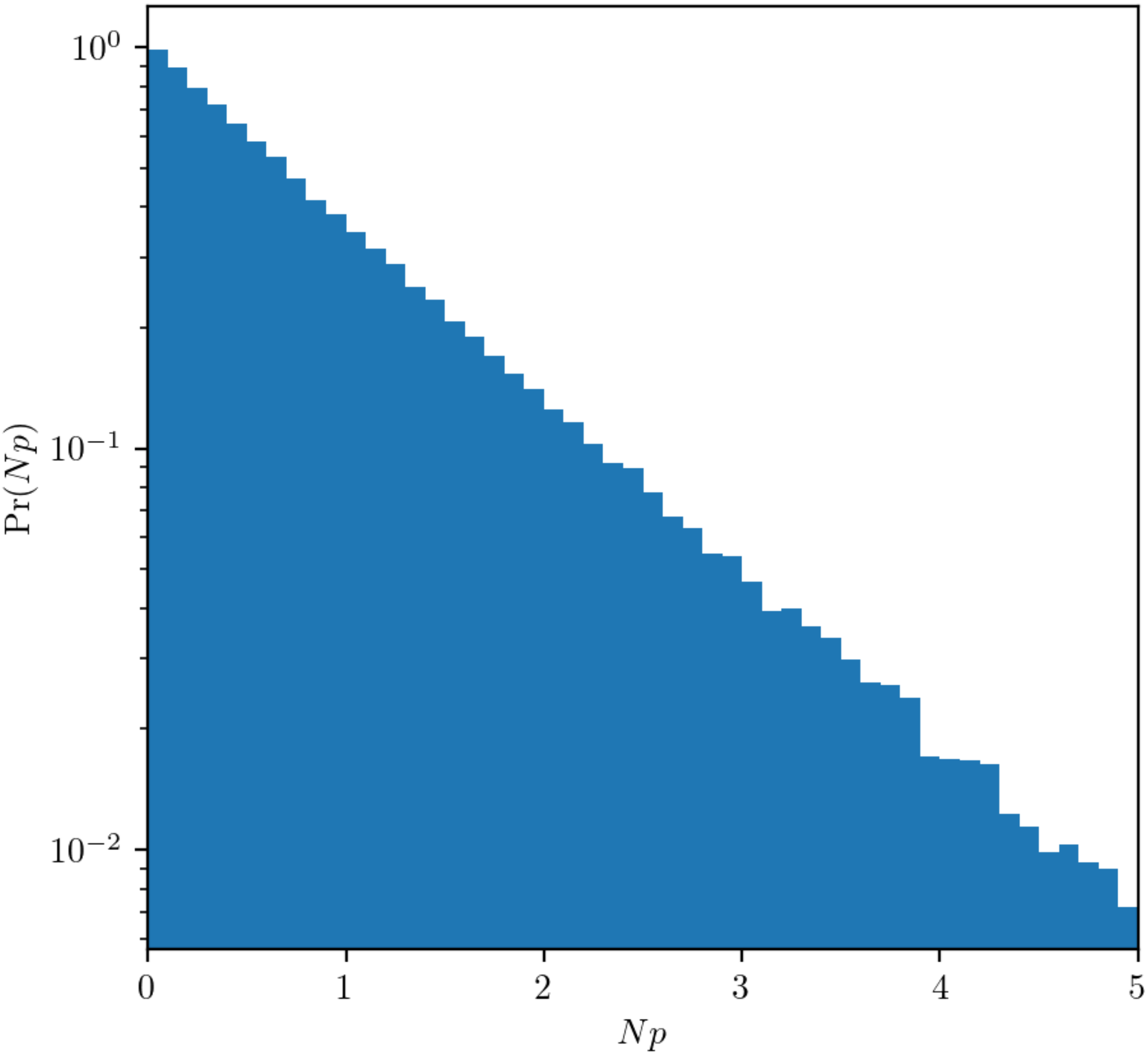

Supplementary Figure 3: The distribution of measurement probabilities from the 200,000 amplitudes calculated for Bristlecone-70 circuits with depth 1+28+1 . It closely matches the Porter-Thomas form Ne^{-Np}.

memory were used to support a double-precision simulation of a 45-qubit quantum circuit on the Cori II supercomputer. The simulation makes use of highly optimized CPU kernels, which are not applicable to Summit, a supercomputer with most of its computational power in GPUs. Furthermore, Pednault et al. [10] scales the runtime down based on LINPACK benchmark figures for Cori II and Summit, making two implicit assumptions. First, the CPU kernels in Häner and Steiger [11] that are highly optimized for the task of quantum simulation are portable to GPUs with no loss in efficiency. Second, the LINPACK benchmark figures, which allow the user to choose the most suitable problem size for a given machine, are applicable to the specific task that Häner and Steiger [11] performs on a single node: simulating up to 32 local qubits. This is problematic given that each socket (the Summit equivalent of a node in Pednault et al. [10]) only has $16 \times 3 = 48$ GiB of GPU memory among its 3 GPUs, while data transfer to and from the GPU can be quite expensive.

For comparison, we estimated the theoretical runtime of our simulator for the 20-cycle quantum superiority task under two analogous assumptions. First, a further optimized implementation should achieve a GPU FLOPS efficiency comparable to

Cotengra, a simulator fundamentally similar to ours. In 18- and 20-cycle experiments on V100 GPUs, we observe a GPU efficiency in excess of 90% with respect to the single-precision performance figure of 15.7 teraFLOPS. Second, we assume that tensors of rank 32 can be contracted on GPUs, presumably using out-of-core memory access, without a significant loss in efficiency. We would then only need to slice enough indices to decrease the contraction width to 32, for which we found a contraction scheme with total cost $10^{18.6}$. Combining both assumptions yields the estimate

$$\frac{(8 \times 10^{18.6})\text{ FLOPs/sample}}{15.7 \times 10^{12}\text{ FLOPS} \times 90\% \times 27648} \times 2000\text{ samples} \approx 1.63 \times 10^{5}\text{ seconds} \approx 1.89\text{ days}.$$

III. CLASSICAL SIMULATION OF THE RANDOM CIRCUIT SAMPLING TASK

In this section, we focus on the random quantum circuits executed on the 53-qubit Sycamore quantum chip [9]. This family of random quantum circuits was proposed to demonstrate the so-called quantum superiority. It was claimed that a certain random sampling task based on these random quantum circuits can be efficiently done on the existing Sycamore quantum chip in about 600s, whereas a comparable task takes Summit, one of the most powerful supercomputers in the world, at least 10,000 years. The Sycamore random circuit sampling task then becomes a benchmark for classical simulation algorithms to ensure the soundness of the claim of quantum superiority. We show that our algorithm helps greatly reduce the classical running time for such a task. On the open-source software package ACQDP we developed implementing the order finding algorithm, it is projected that what previously took 10,000 years to achieve can now be done within 20 days.

A. The Sycamore random circuit sampling task

The Sycamore random circuit sampling task [9] is defined as approximate sampling from the circuit illustrated in Figure 4, where single-qubit and two-qubit gates are specified in Section VI B. A quantum circuit U naturally defines a distribution $\mathcal{D}_U$ over bitstrings when all qubits are measured under the computational basis after executing the circuit on the all-zero state: $\mathcal{D}_U(x) := |\langle x|U|0\rangle|^2$. A quantum device would ideally perform a sampling task according to $\mathcal{D}_U$, but the sampling could only be done approximately on real-life devices due to imperfection in manufacturing and execution of the quantum circuit. For noisy quantum devices, a measure is needed to assess the closeness of the output distribution to the ideal Porter-Thomas distribution. The linear cross-entropy benchmarking fidelity (XEB) was used for this purpose. It is defined as $2^n\langle p_I(x)\rangle - 1$, where n is the number of qubits, $p_I(x)$ is the probability of x in the ideal distribution, and the expectation is taken over the output distribution. The XEB is 0 when the output distribution is uniform, and is 1 when the output distribution is ideal. Simplified quantum circuits run on Sycamore achieved an XEB of approximately 0.2% at 20 cycles. It was argued from numerical evidence that the aforementioned random quantum circuits had also achieved an XEB of approximately 0.2%. However, simulating these circuits was estimated to be infeasible, and so this could not be directly verified.

B. Classical simulation of superiority circuits

To translate the quantum circuit sampling task to one that is well-defined on classical computational resources, we focus on unbiased noise approximate sampling, defined in main text Definition 1, as an analog of what has been done on the quantum devices. We regard a classical simulator to behave comparable to the quantum device if it achieves $(\epsilon, 0.2\%)$-UNA sampling with negligible ϵ (rough estimation shows that $\epsilon < 6.4 \times 10^{-31}$ in our algorithm).

Although a quantum circuit can be readily regarded as a tensor network, the task of contracting such a tensor network is often related to computing one or more amplitudes in the final state, which is not exactly the same as an approximate sampling task. However, for the particular problem of Sycamore random circuit sampling, there is a framework [9, 12] that reduces the sampling problem in this case to amplitude calculation. This framework assumes that the outcome distribution of a random quantum circuit is a randomly permuted Porter-Thomas distribution. Under this assumption, we can perform *frugal rejection sampling* on bitstrings by computing the corresponding amplitudes [13]. When the batch size of bitstrings is sufficiently large (chosen in our case to be 64), then with high probability, at least one outcome among the batch will be accepted. It can be estimated from Markov et al. [13] that this framework essentially equates the task of sampling one sample from the ideal distribution to calculating a batch of 64 amplitudes. We can choose the batch to be a state vector on 6 qubits while randomly post-selecting the remaining 47 qubits. In this case, the aggregated result of the amplitudes can be expressed as an open tensor network. This translates the task of sampling from random quantum circuits to the task of contracting a tensor network. Following the observation in Villalonga et al. [8], simultaneously evaluating 64 amplitudes as an open tensor network yields more efficient sampling, as it is not significantly more costly than evaluating a single amplitude as a closed tensor network. By comparison, performing rejection sampling on single amplitude calculations leads to an approximately $10\times$ overhead. For random circuits

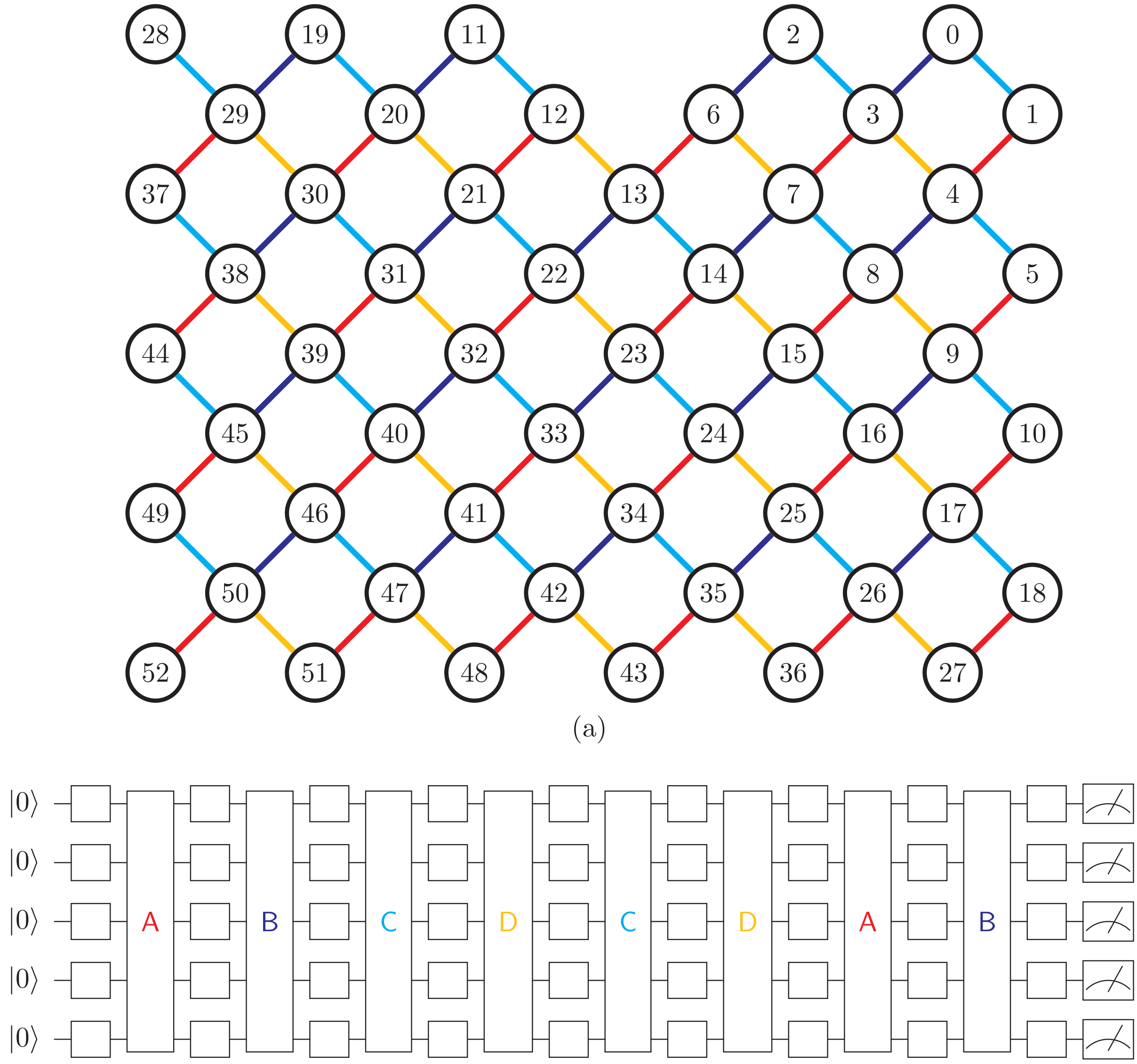

(b)

Supplementary Figure 4: Structure of the 53-qubit random quantum circuits simulated in this work. (a) Layout of the 53 qubits and the two-qubit gates between them. Lines of different colors represent two-qubit gates that appear in different layers. (b) Schematic diagram of an 8-cycle circuit. Each cycle includes a layer of random single-qubit gates (empty squares in the diagram) and a layer of two-qubit gates (labeled A, B, C, or D, and colored according to the two-qubit gates in (a)). For longer circuits, the layers repeat in the sequence A, B, C, D, C, D, A, B. Note that there is an extra layer of single-qubit gates preceding measurement.

with $m = 12, 14, 20$ cycles, we choose the qubits $(0, 1, 2, 3, 4, 5)$ in the upper-right corner, and for $m = 16, 18$ cycles, we choose the qubits $(10, 17, 26, 36, 27, 18)$ in the lower-right corner. This choice is based on the observation that the quantum information only propagates among the selected qubits in the last two layers of the quantum circuit, hence evaluating such a batch of amplitudes should introduce the minimal amount of overhead compared to calculating a single amplitude.

In Pan et al. [14], 2^{21} amplitudes are computed through essentially a single tensor network contraction task, which removes the linear dependence of the number of tensor networks to be contracted with respect to the number of samples. It is a natural question to ask if similar techniques can be translated to our UNA sampling framework to further reduce the time complexity. One major issue of such a technique is that the bit strings sampled from a single batch share a large number of identical bits, a scenario different from the statistical behaviour of a mixture of the Porter-Thomas distribution and a uniform distribution. Therefore, taking more than one bit string from a batch only makes sense when only a few qubits remain closed, which is essentially a full state-vector update. Furthermore, an analysis in Markov et al. [13] indicates that a batch of 64 amplitudes is

enough for a near unity acceptance rate of a single bit string, and further increasing the batch size does not help much in expected number of trials. Therefore, simply increasing the batch size does not help reduce the runtime of our algorithm.

With the above algorithm sampling one near-perfect sample at a time, the task of (ϵ, F)-UNA sampling can be done by randomly mixing uniform bitstrings with the ones that come from the actual sampling routine. The total time for sampling N such samples is than the same as FN "genuine" samples as generating uniform bitstrings can be done efficiently.

C. The superiority tasks for random circuit sampling

The quantum superiority sampling task is described as sampling from a noisy random quantum circuit at a certain fidelity (see Section VIII of [9], here "fidelity" is, in our interpretation, defined through the notion of "linear crossentropy (linear XEB)"). Quantum superiority is then evidenced by statistical analyses that reject the null hypothesis that the quantum device samples with a fidelity $F \leq 10^{-3}$, with a statistical significance of 6σ. As there appears no formal definition of the superiority task, we note, however, that this notion of fidelity, or how well the algorithm can predict the output of a typical quantum random circuit, appear not formally related to the superiority task itself. Rather, the connection relies on assumptions on the behavior of the quantum device. Such a claim is informative for benchmarking purposes, but is not sufficient to establish a comparison between the quantum algorithm and a classical competitors.

To make things precise, we first define the ideal sampling task to be generating i.i.d samples from the ideal distribution $\mathcal{D}_U$. Sampling from an ideal distribution could be infeasibly hard even for the quantum device, due to the many error sources including imperfection of manufacturing, imprecision of control and runtime environmental noise. An experimentally observed noise model is needed so that the sampling task becomes feasible for quantum devices. Different noise models have been proposed in the literature. In Arute et al. [9], the authors proposed a noise model called the *digital error model*, where each gate undergoes an independent depolarization channel, of which the strength only depends on the gate and is time-invariant. It is evidenced qualitatively that the actual quantum device follows the digital error model well for *smaller* and *simpler* quantum systems than the 53-qubit 20-cycle superiority circuits. How well the model applies to the latter family of circuits, which create much more quantum entanglement, remains not directly validated. A simplified noise model, i.e. the real distribution being a probabilistic mixture of the ideal one and the uniform distribution, was then proposed in the same paper based on phenomenological observations, giving rise to the UNA-sampling task defined in Section III B.

The hardness of F-UNA sampling was discussed in Arute et al. [9], but it is explicitly stated that the error model is used for carrying out complexity-theoretic arguments establishing hardness of sampling, and is not meant to be a precise description of the behavior of the quantum device.

The aforementioned task of sampling from a certain family of distributions are difficult to verify without full tomography of the distribution. For the Sycamore quantum chip with 53 qubits, full distribution tomography would require an infeasible amount of samples. Instead, a substitute task called *F-linear XEB benchmarking* is introduced, which can be verified in a sample-efficient way:

Supplementary Definition 1 (F-linear XEB benchmarking)**.** For a quantum circuit U as a unitary on n qubits, the task of F-linear XEB benchmarking is to generate samples $\{x_1, \ldots, x_N\}$ such that

$$\frac{2^n}{N} \sum_i \mathcal{D}_U(x_i) - 1 \geq F.$$

Under the F-UNA noise model, a quantum computer only needs to sample $N = O(F^{-2})$ samples in order for the sample mean to converge close to F, contrary to $\Omega(4^n)$ samples for full tomography when F is significant. In Arute et al. [9], linear XEB has been used as the main benchmarking quantity, and extensive, yet indirect numerical evidence was provided to establish an estimate of linear XEB to be above 0.1% for the Sycamore random circuits.

It is evident that linear XEB benchmarking can be met through UNA sampling; however, the other direction is not true due to the presence of many other candidate distributions satisfying the linear XEB requirement. In Arute et al. [9], two classical candidates are provided in comparison to the quantum device. The qFlex algorithm does rejection sampling based on the Porter-Thomas assumption, and is believed to achieve (ϵ, F)-UNA sampling, where $F = 0.2\%$ and $\epsilon \approx 6.4 \times 10^{-31}$ according to arguments given in Markov et al. [13]. However, due to memory constraints of the qFlex algorithm, only superiority circuits up to $m = 14$ was estimated. The other algorithm, called SFA, is believed to be sampling from a distribution with $\approx 0.2\%$ linear XEB with respect to the ideal distribution, but is not necessarily a UNA sampling. The SFA algorithm is much more efficient compared to the qFlex algorithm, and a 10,000-year estimate is provided for the SFA on the Summit supercomputer.

Our result of 20 days is based on the framework of the qFlex algorithm, and also achieves $(6.4 \times 10^{-31}, 0.2\%)$-UNA sampling. This choice is made in order to establish a comparison with the existing tensor-network-based algorithms. However, the corresponding classical runtime could be further reduced by orders-of-magnitude should linear XEB be the chosen task, as is verified for the quantum device. For example, one could repeatedly output a single high-probability bitstring. This requires computing only one batch in expectation, and can be done in approximately 15 minutes by our algorithm. In fact, recent work

presents an algorithm which can pass the linear XEB test within polynomial time for shallow circuits, undermining the classical difficulty of outputting samples with high linear XEB [15].

Such an attack using a singleton distribution may be trivial and could be remedied by slightly augmenting the linear XEB benchmarking task, but it exposes the complications resulting from the ambiguity in the current definition. We also note that such remedies might not be obvious: the sampling task should be made hard enough to restrict classical shortcuts for reducing runtime, but at the same time, not too restrictive that quantum devices cannot achieve it. In fact, a further Kolmogorov-Smirnov test, can be regarded as a candidate additional test to verify the superiority task [9]. Numerical experiments show that this test can also be easily passed using classical means with a single batch of 64 amplitudes in 15 minutes.

A very recent result by Pan et al. [14] claimed to have completed the Sycamore random circuit sampling task, by producing 1 million distinct bistrings within 5 days using 60 Nvidia A100 GPUs, achieving a linear XEB value of ~ 0.739. This is made possible by two main ingredients presented in the paper:

- The main computation performed in Pan et al. is a contraction of a tensor network with 21 open edges. Alongside adopting several methodologies from this paper, including index slicing and GPU-oriented runtime optimization, the authors developed their own heuristics for contracting tensor networks, including the *big-head* contraction order finding, which helped greatly increase the efficiency of contracting tensor networks with many open edges.
- While our work focuses on generating i.i.d. samples from the mixture of the uniform distribution and the ideal distribution defined by a certain random circuit, such an interpretation is only one of the possible ones associated to the random circuit sampling task. In fact, Pan et al. took a different interpretation and only required the output bitstrings be distinct. As a result, all the bitstrings share a 32-bit all-zero prefix, which made the computational task much easier compared to the sampling task we pursue in this paper. While such a result might not be directly comparable with the efficiency of our implementation dedicated to achieving the UNA sampling, it highlights the ambiguity of the quantum superiority task itself.

There may be room to debate what properties a precise definition of a superiority task should have. For the least, such a definition should allow the possibility of a refutation by a classical algorithm. Furthermore, it'd be desirable that such a refutation can be verified easily.

D. Simulation Cost of Random Quantum Circuit Sampling

We implemented the tensor network contraction algorithm as an open-source software ACQDP, available on GitHub[38]. Here we benchmark our simulator, the ACQDP, on the aforementioned random quantum circuits. The circuit files we used are drawn from the data supplement to Arute et al. [9] and are available at a public Dryad repository [7]. We chose the latest version (Jan.23) that includes circuit files with 12, 14, 16, 18, and 20 cycles.

In the preprocessing step for generating a contraction order, we allow for 50 iterations of `CMA-ES` for parameter optimization and choose the number of local optimization iterations before, between, and after slicing to be 20, 20, and 50, respectively. The generated contraction order is then distributed to the computational nodes. We run each circuit 10 times and choose the best contraction order. The time for each run ranges from 1 hour up to 8 hours depending on the circuit size. Figure 2 shows the contraction cost for each run. Detailed information about our cluster architecture can also be found in Section II in the Supplementary Information.

IV. APPLICATION TO QUANTUM ALGORITHM DESIGN

In this section, we apply our algorithm to tensor networks in the context of near term quantum algorithm design. Specifically, we use our tensor network contraction algorithm to characterize graph isomorphism classes inspired by the Quantum Approximate Optimization Algorithm (QAOA).

A. The QAOA

Quantum Approximate Optimization Algorithm (QAOA) was first developed by Farhi, Goldstone and Gutman [16] to solve combinatorial optimization problems, and was inspired by the quantum adiabatic algorithm. Unlike the quantum adiabatic algorithm, the QAOA uses shallow quantum circuits and classical feedback, making it a prime example of a hybrid quantum-classical algorithm readily applicable to near-term quantum devices. In this section, we are only interested in the QAOA energy function defined below as a candidate characterization of the graph isomorphism classes.

The QAOA aims to optimize via a quantum variational approach over combinatorial problems of the form $C : \{0,1\}^n \to \mathbb{R}$, which can be decomposed as a sum of local clauses $C = \sum_{i=1}^{m} C_i$ each acting only on a small number of bits. Regarding the objective function $C : 2^n \to \mathbb{R}$ as a local Hamiltonian $\hat{C} = \sum_x f(x)|x\rangle\langle x| = \sum_{j=1}^{m} \hat{C}_j$, QAOA takes the ansatz that the state

$$|\vec{\gamma}, \vec{\beta}\rangle = e^{-i\beta_p \hat{B}} e^{-i\gamma_p \hat{C}} \cdots e^{-i\beta_1 \hat{B}} e^{-i\gamma_1 \hat{C}} (|+\rangle)^{\otimes n} \tag{2}$$

defined by the mixing operator $\hat{B} = \sum_{i=1}^{n} X_i$ and the angle sequences $\vec{\gamma}, \vec{\beta} \in \mathbb{R}^p$ approaches the ground state of $\hat{C}$ with carefully chosen parameters $\vec{\gamma}, \vec{\beta}$, even with a small number of layers p. In the case that the coefficients in the objective function C takes integer values, it is sufficient to restrict to $\vec{\gamma}, \vec{\beta} \in [0, 2\pi]^p$. Since

$$e^{-i\beta \hat{B}} = \bigotimes_{j=1}^{n} e^{-i\beta X_j}$$

can be decomposed into individual X-rotations, and

$$e^{-i\gamma \hat{C}} = \prod_{j=1}^{m} e^{-i\gamma \hat{C}_j}$$

can be decomposed into commuting unitary operations each acting on a small number of qubits, a state $|\vec{\gamma}, \vec{\beta}\rangle$ can be readily prepared using a quantum circuit.

The QAOA energy function with p layers is defined as follows:

$$F_p(\vec{\gamma}, \vec{\beta}) := \langle \vec{\gamma}, \vec{\beta} | \hat{C} | \vec{\gamma}, \vec{\beta} \rangle.$$

The operational meaning of $F_p(\vec{\gamma}, \vec{\beta})$ is the expectation value of $C(Z)$, where the random string Z comes from measuring the quantum state $|\vec{\gamma}, \vec{\beta}\rangle$ under the computational basis. The QAOA energy function indicates the performance of the QAOA algorithm itself, and is commonly used as the objective function for finding good angle sequences $\vec{\gamma}, \vec{\beta}$ via a classical optimizer.

B. Graph Isomorphism

Two graphs $G = (V_G, E_G)$ and $H = (V_H, E_H)$ are called *isomorphic* if there is a one-one correspondence f of the vertices of G to the vertices of H that preserves the edge connection: $(u, v) \in E_G \Leftrightarrow (f(u), f(v)) \in E_H$.

To map the problem of graph isomorphism to the QAOA, we associate with each graph $G = (V, E)$ the cut function defined over arbitrary bipartition of its vertices: $M_G(x) = \sum_{u,v \in V, (u,v) \in E} x_u \oplus x_v$, from which QAOA energies can be defined accordingly with respect to the Hamiltonian $\hat{C} = \sum_{(u,v) \in E} Z_u Z_v$. Two isomorphic graphs always give the same QAOA energies under the same fixed angle sequence $(\vec{\gamma}, \vec{\beta})$ (the optimization step does not play a role here). One can potentially discover non-isomorphism with the algorithm:

1. Pick $(\vec{\gamma}, \vec{\beta}) \in [0, 2\pi]^{2p}$ uniformly at random, for predetermined number of layers p.
2. Compute $E_1 = F_p^{(1)}(\vec{\gamma}, \vec{\beta})$ and $E_2 = F_p^{(2)}(\vec{\gamma}, \vec{\beta})$, where $F_p^{(1)}$ and $F_p^{(2)}$ are the QAOA energy functions associated with the CUT function of the two graphs.
3. Output "yes" if $E_1 = E_2$, otherwise output "no." (Repeat the procedure for higher certainty.)

Conjecture 1 ([17])**.** For any $n \in \mathbb{N}_+$, for sufficiently large p, with probability 1 over all angle sequences in $[0, 2\pi]^{2p}$ the above algorithm gives different energy values when G_1 and G_2 on n nodes are non-isomorphic.

Even when the conjecture holds, it is not sufficient to put the graph isomorphism problem in quantum polynomial time, since the difference between the energy values can be exponentially small.

Graph isomorphism has been extensively studied in classical complexity theory, where a breakthrough result of László Babai, obtained in 2016, ensures that graph isomorphism is in $\exp(C(\log n)^c)$, i.e. in quasi polynomial classical time [18]. There have been several attempts to construct quantum operators from walks to identify isomorphism classes of graphs. A promising recent attempt is that of Kamil Brádler, Shmuel Friedland, Josh Izaac, Nathan Killoran and Daiqin Su of Xanudu. The authors have found out that their boson sampling experiments always distinguishes between two non-isomorphic graphs [19]. Unfortunately, the argument that proves this fact does not give an efficient bound on the energy gap.

C. Simulation and Results

We use our tensor network contraction algorithm to compute the energy values $F_p(\vec{\gamma}, \vec{\beta}) = \langle \vec{\gamma}, \vec{\beta} | \hat{C} | \vec{\gamma}, \vec{\beta} \rangle$. Several observations are applied in order to make the simulation easier, which has been summarized in Figure 5:

- A straightforward observation to accelerate the simulation is the fact that the operator $\hat{C}$ does not have a simple tensor structure; instead each of its summands are small tensors and it could be easier to compute each term $\langle \vec{\gamma}, \vec{\beta} | \hat{C}_i | \vec{\gamma}, \vec{\beta} \rangle$ individually.
- The idea of lightcone is to cancel out circuit components that commute through the local Hamiltonian term [16]. In other words, only qubits and gates that lie in the "lightcone" of the Hamiltonian term need to be taken into account. This greatly simplifies the tensor networks, especially in cases where the Hamiltonian is sparse and the depth of the QAOA is relatively small.
- The energy function is not only invariant under permutation of vertices; rather, an individual energy term $E_{(i,j)}(\vec{\gamma}, \vec{\beta}) = \langle \vec{\gamma}, \vec{\beta} | Z_i Z_j | \vec{\gamma}, \vec{\beta} \rangle$ associated with an edge (i, j) always equals to $E_{(i',j')}(\vec{\gamma}, \vec{\beta})$ if there is an automorphism of the graph that maps (i, j) to (i', j'), as they correspond to identical tensor networks. This is in particular useful when the graph considered exhibits multiple symmetries.

V. APPLICATION TO QUANTUM ERROR CORRECTION

In this section, we apply our tensor network contraction algorithm to study the logical errors occurring in quantum error correction. Specifically, we consider a quantum memory experiment on a surface code with 17 qubits, called the Surface-17 for short, in the presence of a practical noise model first proposed in O'Brien et al. [20], and an additional crosstalk model which was not considered before in this context. With the tensor network contraction algorithm, we are able to extend the simulation of exact performances of optimal decoders into the fault-tolerant regime, where we can accurately compute each component of the logical error, without ever resolving to randomized schemes that might require a prohibitive amount of samples to get a relatively accurate result. The tensor network also allows us to study variants of the noise model including different scheduling of physical gates and variations of the ordinary surface code, such as the XZZX code [21].

A. Surface code and logical memory experiment

a. Surface-17 A surface code is a stabilizer code defined on a square grid. Here we consider the surface code defined on a 3×3 grid, which consists of 9 physical qubits. To realize the code in practice, ancilla qubits must be introduced in order to extract the classical syndromes used for error correction. The 9 physical qubits, together with 4 ancilla qubits for X stabilizer measurements and 4 ancilla qubits for Z stabilizer measurements form the Surface-17, illustrated in Figure 6.

b. Logical Memory Experiment In this section, we report the simulation results for a Surface-17 simulation at the level of the logical quantum memory. Each experiment consists of k rounds of noisy syndrome extraction, and one round of noiseless syndrome extraction together with a trivial decoder for this round. The goal of the trivial decoder is to perform simple corrections so that the final state lies in the code space. In particular, we choose a weight-1 pure error (see Chapter 6.3 of Lidar et al. [22]) for each stabilizer we measure. If the measurement outcome of a stabilizer in the final round is -1, we noiselessly apply the corresponding pure error to correct it. The exact assignments of pure errors are not important because we will perform the actual decoding, which is insensitive to these assignments, afterwards. As a result, we have a channel which leaves the code space invariant, and to each syndrome we must assign a logical Pauli correction. The full logical channel is then obtained by choosing the optimal logical Pauli correction conditioned on each syndrome, and summing up over all syndromes.

For simplicity, we focus on the channel restricted to the code space. We use the initial state

$$|\Psi\rangle = |0\rangle_{\text{logical}} |0\rangle_{\text{extra}} + |1\rangle_{\text{logical}} |1\rangle_{\text{extra}},$$

which is a maximally entangled state between the logical qubit and an additional virtual qubit. The extra qubit is only used to extract the logical channel via the Choi–Jamiołkowski isomorphism. Note that the resulting simulation is unphysical in the sense that it does not represent a real experiment, but nonetheless gives information quantifying how damaging different errors can be.

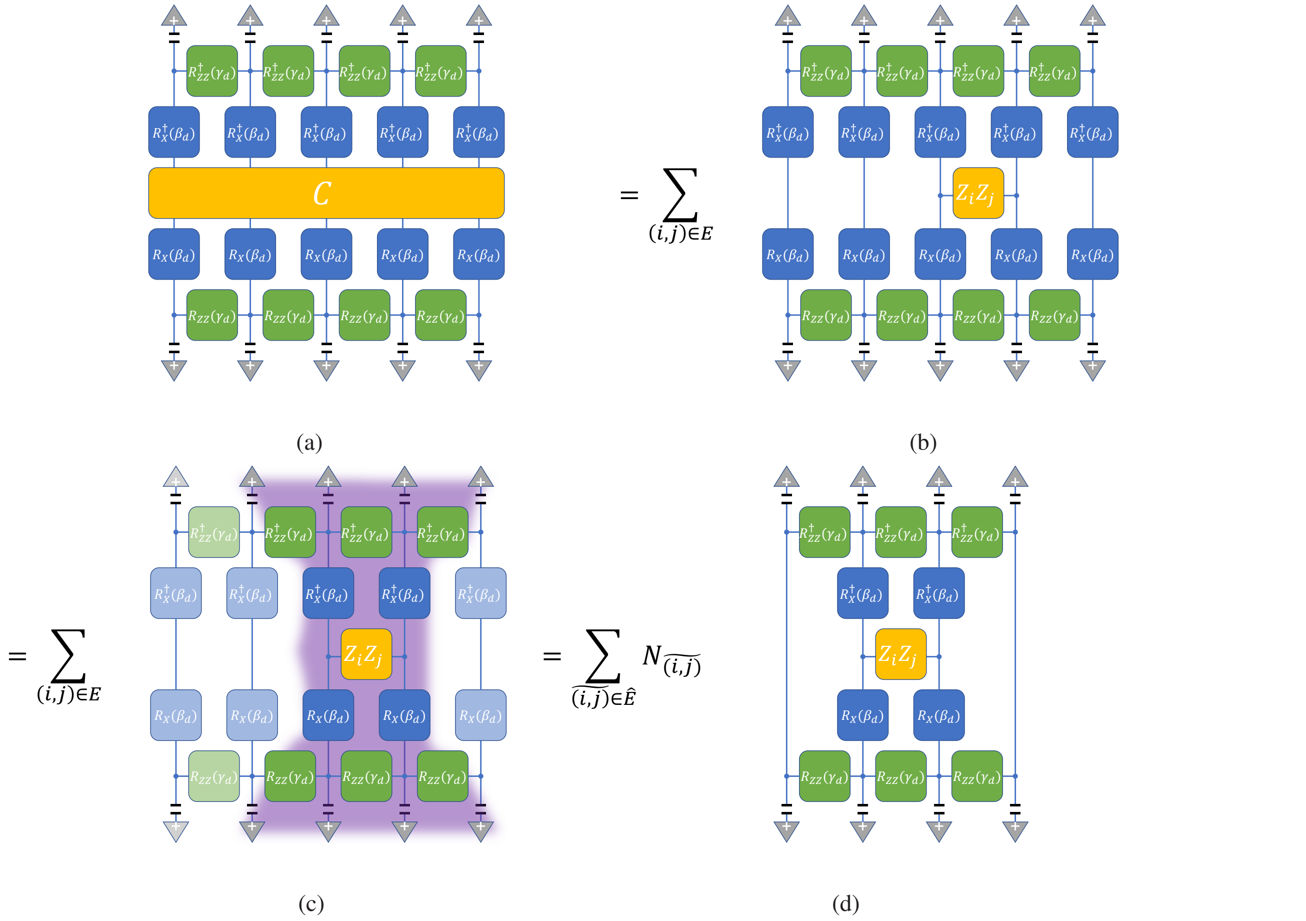

Supplementary Figure 5: Illustration of simplifications of QAOA energy queries using classical simulation, assuming each clause corresponds to a ZZ operator as in the case of MAX-CUT. (a) The QAOA energy query illustrated as a tensor network. Note that the Hamiltonian term C is usually a large tensor. (b) Breaking down the tensor network into summations of energy terms associated with individual clauses for possible speed-up. (c) Any component outside of the purple region (the lightcone) of the Hamiltonian term, depicted transparent, can be cancelled out to simplify the tensor networks. (d) Energy associated with edges isomorphic to each other are always identical and can be grouped together and evaluate only once, avoiding redundant calculation. Here $\widetilde{(i,j)}$ stands for the isomorphic class of the edge (i,j) and $N_{\widetilde{(i,j)}}$ stands for its multiplicity.

c. Optimal Decoder We study the performance of the Surface-17 under the optimal decoder. An optimal decoder, upon obtaining a bitstring of syndromes S, computes classically the conditional logical state ρ_S conditioned on the syndrome bits S on the composite system of the logical qubit and the entangled extra qubit. It then apply the best Pauli correction σ_S selected from $\{I, X, Y, Z\}$ on the logical system to maximize the overlap between the corrected state $\rho'_S = (\sigma_S \otimes I)\rho_S(\sigma_S^\dagger \otimes I)$ and the Bell State $|\Psi\rangle = |0\rangle_{\text{logical}}|0\rangle_{\text{extra}} + |1\rangle_{\text{logical}}|1\rangle_{\text{extra}}$. The final logical channel, after applying the optimal decoder, is the probabilistic average of the corrected ρ'_S over all syndromes.

The optimal decoder is not an efficient one, as it involves heavy computation to simulate the logical conditional state ρ_S in order to pick the Pauli correction. In fact, there have been multiple attempts on designing efficient near-optimal decoders including the minimum-weight perfect matching [23, 24] and union-find algorithms [25–29]. The reason for our choice is two-fold. First, optimal decoders, despite being ultimately unscalable, are within reach for experiments on the Surface-17. Second, using the optimal decoder in the simulation allows us to focus on the quantum hardware to determine the effects of each hardware parameter on the performance of quantum error correction, so that the results can best guide the demonstration of a fault-tolerance pseudothreshold.

d. Pauli Transition Matrix Instead of focusing on a specific quantity characterizing the error rate, we report the whole logical channel in the form of a Pauli transition matrix (PTM). The PTM of a single-qubit channel $\mathcal{C}$ is a 4×4 real matrix defined as

$$P(\mathcal{C})_{ij} = \frac{1}{2}\text{Tr}[\sigma_i \mathcal{C}(\sigma_j)],$$

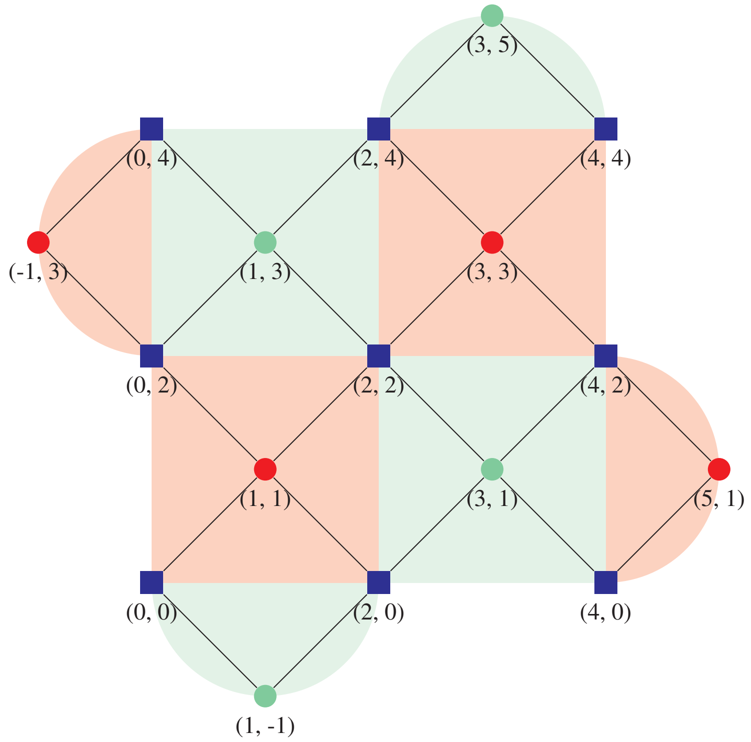

Supplementary Figure 6: Illustration of the Surface-17. The blue squares (e.g. at $(2,2)$), red circles (e.g. at $(3,3)$), and green circles (e.g. at $(1,3)$) represent the data, X-, and Z-ancilla qubits, respectively. The shaded red and green regions represent the X- and Z-type stabilizers, respectively. Two-qubit interactions only exist between connected qubit pairs.

where $\sigma_0, \sigma_1, \sigma_2, \sigma_3 = I, X, Y, Z$, respectively. Note that the first row of any PTM corresponding to a completely positive and trace preserving (CPTP) map is always $(1,0,0,0)$ since

$$\mathrm{Tr}[\sigma_0 \mathcal{C}(\sigma_j)] = \mathrm{Tr}[\mathcal{C}(\sigma_j)] = \mathrm{Tr}[\sigma_j] = \begin{cases} 1 & \text{if } j = 0; \\ 0 & \text{if } j = 1,2,3. \end{cases}$$

The other 12 entries in the matrix represent the degrees of freedom, where roughly, the first column indicates non-unital shifts, the diagonal terms indicate dephasing errors and the off-diagonal terms indicate coherent errors (See main text Figure 5a). Moreover, the phase-flip error rate p_{phase} and bit-flip error rate p_{bit} can be directly deduced from the corresponding PTM entries. In main text Figure 5a, we denote by $p_{\text{bit}}, p_{\text{phase}}$ and p_Y the probability that randomly prepared logical states $\{|0\rangle, |1\rangle\}(\{|+\rangle, |-\rangle\}, \{|+i\rangle, |-i\rangle\})$ are measured opposite after the channel respectively.

B. Experimental Error Model

Whereas O'Brien et al. [20] uses Pauli transfer matrices to represent error channels, we will use Choi matrices, as they are closer to the tensor representations used in our program.

1. *Idle Error*

The error on an idling qubit is described by the standard amplitude-phase damping model, which as the name suggests, consists of two components:

- Amplitude damping, which causes a qubit A to relax from the excited state $|1\rangle_A$ to the ground state $|0\rangle_A$. When idling for some time t, the excited state has a probability p_1 to emit a photon and decay to the ground state. We can assume that the environment electromagnetic field is initialized in the vacuum state $|0\rangle_E$, and the emission of a photon is a unitary process that changes the environment to $|1\rangle_E$.

  This evolution can be thus described as follows:

– Adding an ancillary qubit $|0\rangle_E$.
– Apply a unitary such that

$$|00\rangle_{AE} \mapsto |00\rangle_{AE} \quad \text{and} \quad |10\rangle_{AE} \mapsto \sqrt{1-p_1}|10\rangle_{AE} + \sqrt{p_1}|01\rangle_{AE}.$$

– Discard the ancilla.

- Phase damping, which causes the qubit to dephase into the computational basis $\{|0\rangle_A, |1\rangle_A\}$. This occurs due to interactions that are weak compared with the system energy but strong compared with the environment energy. These are not strong enough to cause the qubit to flip from the excited state to the ground state or vice versa, but may cause the environment state to change depending on the qubit state.

  This evolution can be described as follows:

  – Adding an ancillary qubit $|0\rangle_E$.
  – Apply a unitary such that

$$|00\rangle_{AE} \mapsto |00\rangle_{AE} \quad \text{and} \quad |10\rangle_{AE} \mapsto \sqrt{1-p_\phi}|10\rangle_{AE} + \sqrt{p_\phi}|11\rangle_{AE}.$$

  – Discard the ancilla.

In the Choi matrix representation:

$$C_{\Lambda_{T_1}} = \left[\begin{array}{cc|cc} 1 & 0 & 0 & \sqrt{1-p_1} \\ 0 & 0 & 0 & 0 \\ \hline 0 & 0 & p_1 & 0 \\ \sqrt{1-p_1} & 0 & 0 & 1-p_1 \end{array}\right] \quad \text{and} \quad C_{\Lambda_{T_\phi}} = \left[\begin{array}{cc|cc} 1 & 0 & 0 & \sqrt{1-p_\phi} \\ 0 & 0 & 0 & 0 \\ \hline 0 & 0 & 0 & 0 \\ \sqrt{1-p_\phi} & 0 & 0 & 1 \end{array}\right].$$

The error parameters $1-p_1$ and $1-p_\phi$ decay exponentially with the idle duration t:

$$1-p_1 = e^{-t/T_1} \quad \text{and} \quad 1-p_\phi = e^{-t/T_\phi}.$$

Note that these channels commute with each other, and also satisfy

$$\Lambda(t_1) \circ \Lambda(t_2) = \Lambda(t_1+t_2).$$

In fact, the amplitude- and phase-damping channels model more than just idling. In a superconducting quantum device, amplitude and phase damping occurs on qubits no matter what operations are being applied. As a result, even though every quantum operation takes a certain duration, many can be modeled as happening instantaneously at a single point of time (usually the middle of its time interval), with the amplitude and phase damping channels applied both before and after. Any operation-specific error channel can also be applied at the same time point. Below, we will focus on the operation-specific errors only.

2. $R_y\,(\pm\pi/2)$ *Gates*

Following O'Brien et al. [20], the gate-specific error of $R_y(\pm\pi/2)$ gates is modeled as depolarizing noise that shrinks the Bloch sphere more in the x-z plane and less along the y axis. In the Choi matrix representation:

$$C_{\text{dep}} = \left[\begin{array}{cc|cc} 1-p_{\text{plane}}/2 & 0 & 0 & 1-p_{\text{plane}}/2-p_{\text{axis}}/2 \\ 0 & p_{\text{plane}}/2 & -p_{\text{plane}}/2+p_{\text{axis}}/2 & 0 \\ \hline 0 & -p_{\text{plane}}/2+p_{\text{axis}}/2 & p_{\text{plane}}/2 & 0 \\ 1-p_{\text{plane}}/2-p_{\text{axis}}/2 & 0 & 0 & 1-p_{\text{plane}}/2 \end{array}\right].$$

3. *CZ Gates*

In O'Brien et al. [20], the gate-specific error of CZ gates is modeled as a single-qubit phase error ("flux noise") that only applies to the qubit with higher frequency (whose frequency must be shifted away from its "sweet spot" to enable the interaction), along with a two-qubit phase error due to flux noise.

Other than single-qubit errors as the qubits are idling, additional errors during the CZ gate are "quasi-static", meaning that for each pair of coupled qubits, the phase fluctuations $\delta\phi$ and $\delta\phi_{2\text{Q}}$ are considered constant throughout one run of the circuit, but vary randomly over different runs.

In this article, we choose not to include the quasi-static flux noise mentioned in the Appendix D and E in O'Brien et al. [20], because such low-frequency noises can be compensated by rapid qubit calibrations or spin-echo-like noise canceling techniques [30, 31].

4. Measurement Error

The measurement of a qubit is implemented in two steps. In the first step, photons are introduced into a readout resonator, dephasing the qubit completely into the computational basis and allowing the measurement result to be read out. In the second step, the qubit is left idling for a period of time, allowing the photons to deplete from the resonator.

Although measurement is modeled as a "butterfly gate" in O'Brien et al. [20], they also note that the parameters observed experimentally can be well explained by simply assuming that the measurement happens instantaneously at the halfway point of the measurement duration, and that the result is subject to a classical declaration error ϵ_{RO} that is independent of the outcome.

Another way measurement can cause errors is through leftover photons in the resonator. Even after photon depletion, there will still be a small number of photons remaining in the cavity, causing the qubit to dephase. The mechanism for this dephasing is complicated and is related to the current state of the qubit, but in this particular circuit, its effect in the time interval $[t_1, t_2]$ can be quantified as

$$p_{\phi,\text{photon}} = \exp\left(2\chi\alpha(0)\exp(\kappa(t_m - t_g)) \times \left[\frac{e^{-\kappa t}}{4\chi^2+\kappa^2}[-\kappa\sin(2\chi t) - 2\chi\cos(2\chi t)]\right]_{t_1-t_g}^{t_2-t_g}\right),$$

where t_m is the start time of the measurement period, t_g is the time it takes to rotate the qubit from a computational basis state to a Hadamard basis state during an $R_y(-\pi/2)$ gate, and κ and χ are constant parameters. The error channel resulting from this dephasing is similar to the phase damping channel Λ_{T_ϕ} above, with p_ϕ replaced by $p_{\phi,\text{photon}}$ [20].

5. Crosstalk

We will consider crosstalk effects between neighboring qubits. To clarify, we will only consider the crosstalk effect caused by stray 2-qubit interactions. For example, in DiCarlo et al. [32], it is pointed out that at the flux sweet spot, the ZZ coupling strength is between 0.1% and 1% of the strength at the 2-qubit operating point. We include the following crosstalk Hamiltonian

$$H_{\text{xtalk}} = k\sum_{(i,j)} Z_i Z_j = k\sum_{(i,j)}(-I + Z_i + Z_j + 4|11\rangle\langle 11|_{ij}), \tag{3}$$

where i, j correspond to neighboring qubits. For simplicity, we assume that the crosstalk strength is uniform and time-independent, which may not be the case in real experiments. In Figure 7, we use CPHASE gates to simulate the effects of crosstalk. These CPHASE gates correspond to the term $4k|11\rangle\langle 11|$ in Equation (3). From Equation (3), we can see that CPHASE and e^{ikZZ} are equivalent up to some single qubit Z-rotations. Whether one should use CPHASE or the e^{ikZZ} gate to model simulations mostly depends on how one calibrates the qubit frequencies.

There are two motivations for using a few instantaneous gates to simulate the effect of crosstalk, which is by nature continuous in time. The first motivation is to turn the task of solving Lindblad equations into tensor network contraction of gate-based quantum circuits, which consumes significantly less computational resources. Another motivation more relevant to physical experiments is to measure the extent to which the effects of crosstalk can be compensated for by slightly manipulating the original measurement circuit. In fact, the circuit in Figure 7 already takes this compensation into account.

Note that we will only consider crosstalk noise inside the CZ-region in Figure 7. This is because the CZ-regions occupy the majority of the measurement rounds.

6. Parameters

We adopt the same set of parameters as in O'Brien et al. [20]. In addition, we denote the crosstalk strength by k, which we newly include into the noise model. For the sake of readability, we summarize these parameters in Table 2.

C. Simulation Techniques

a. Specification of the tensor network We simulate the performance of the optimal decoder under the tensor network framework. For the experiment with $k+1$ cycles, an exact computation of the logical error channel requires enumeration over all possible syndrome outcomes $S \in \{0,1\}^{8(k+1)}$ to compute the individual conditional states ρ_S. This overhead can be partially resolved by simultaneously evaluating all the subnormalized logical Choi states together as an aggregated tensor, $\bigoplus_{S\in\{0,1\}^{8(k+1)}} \Pr[S]\cdot\rho_S$. Operationally, this quantity corresponds to the composite classical-quantum state of the syndromes

Category	Parameter	Symbol	Value
scheduling / idle error [Section V B 1]	Qubit relaxation time	T_1	$30\mu s$
scheduling / idle error [Section V B 1]	Qubit dephasing time	T_ϕ	$60\mu s$
scheduling / idle error [Section V B 1]	Single-qubit gate time	$T_{g,1Q}$	20ns
scheduling / idle error [Section V B 1]	Two-qubit gate time	$T_{g,2Q}$	40ns
scheduling / idle error [Section V B 1]	Coherent step time	τ_c	200ns
scheduling / idle error [Section V B 1]	Depletion time	τ_d	300ns
scheduling / idle error [Section V B 1]	Fast depletion time	$\tau_d^{(fast)}$	100ns
scheduling / idle error [Section V B 1]	Measurement time	τ_m	300ns
$R_y(\pm\pi/2)$ gates [Section V B 2]	In-axis rotation error	p_{axis}	10^{-4}
$R_y(\pm\pi/2)$ gates [Section V B 2]	In-plane rotation error	p_{plane}	5×10^{-4}
measurement error [Section V B 4]	Fast measurement time	$\tau_m^{(fast)}$	100ns
measurement error [Section V B 4]	Readout infidelity	ϵ_{RO}	0.15%
measurement error [Section V B 4]	Photon relaxation time	$\frac{1}{\kappa}$	250ns
measurement error [Section V B 4]	Dispersive shift	$\frac{\chi}{\pi}$	-2.6 MHz
crosstalk [Section V B 5]	Crosstalk strength	k	0.03

Supplementary Table 2: Experimental parameters used in our simulation

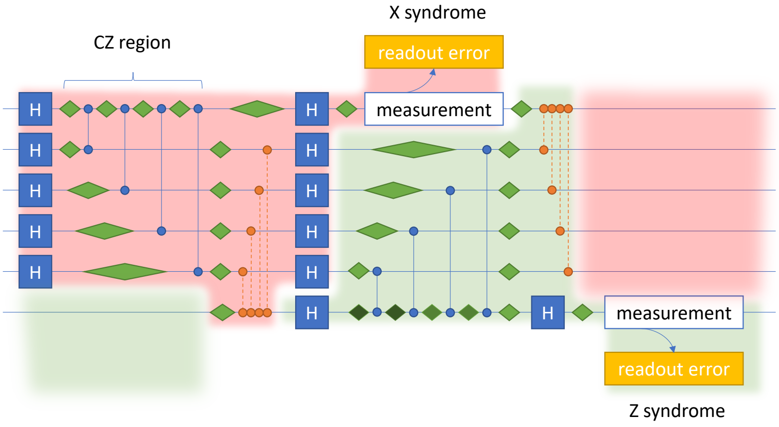

(a) The gate-based noise model. The areas shaded red and green represent respectively the X- and Z- syndrome extraction subroutines. The diagram shows a round of X-syndrome extraction followed by a round of Z-syndrome extraction. Notice that the measurement part of one subroutine overlaps with the CZ gates of the next one. The noise can be roughly classified into four parts: the idling errors (green), the gate-specific errors (blue), the readout errors (yellow) and crosstalk (orange). The dark green diamonds represent amplitude-phase damping channels with an additional phase damping from photon decay. This diagram does not reflect the actual model in the sense that no four data qubits are adjacent to the same X- and Z-type ancilla qubits simultaneously, but we present it this way for the sake of simplicity.

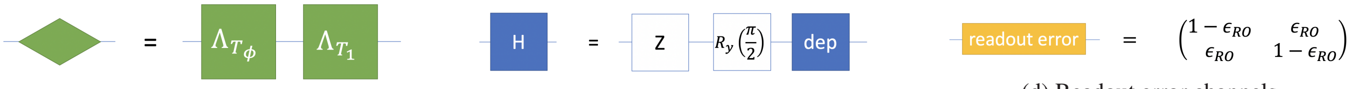

(b) Idling errors

(c) Noisy Hadamard gates

(d) Readout error channels

Supplementary Figure 7: An illustration of the gate-based noise model

together with the final density matrix of the two remaining qubits. See Figure 8 for an illustration of the tensor network representing the c-q state. Once this big tensor is obtained, one can evaluate the optimal Pauli corrections for each syndrome outcome, and compute the aggregated optimal logical channel.

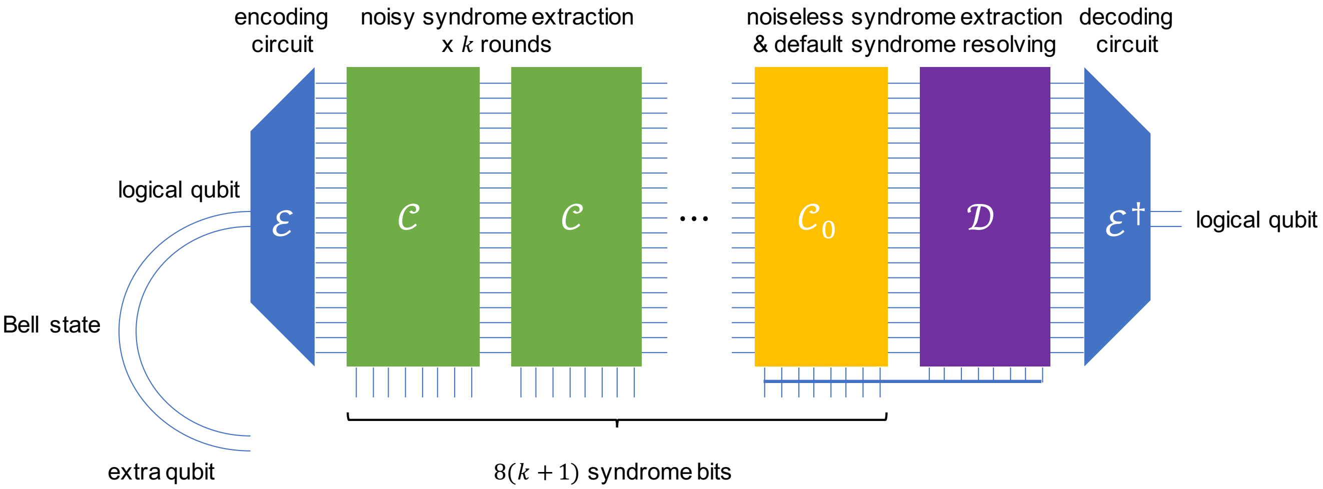

Supplementary Figure 8: Illustration of the tensor network being contracted. The blue lines with unattached endpoints represent open edges of the tensor network. We first prepare the logical and the extra qubit in the Bell state. The logical qubit is then encoded into physical qubits according to the Surface-17, which then goes through k rounds of noise syndrome extraction cycles. The qubits then goes through a cycle of noiseless syndrome extraction, followed by a syndrome resolving phase projecting the qubits into the code subspace. Finally, the physical qubits goes through the decoding circuit to get back the logical qubit. The open edges include $8(k+1)$ syndrome bits, and 4 bits representing the density matrix of the joint state of the logical qubit and the extra qubit. Note that the noise model is only relevant in the k syndrome extraction cycles.

Apparently, the size of the outcome from the tensor network contraction is 2^{8k+12}, and it would grow quickly infeasible to obtain from $k = 3$. Here we report results corresponding to $k = 1$ and 2, as both tasks can still be easily simulated.

D. Discussion

There have been numerous results focusing on simulating the Surface-17 under physically motivated noise models [20, 33–36]. Although Pauli errors and some restricted families admit more efficient classical simulation techniques [35, 36], more accurate characterization of Surface-17 under general noise models often resolve to exact calculation of quantum states outside of the stabilizer formalism, such as density matrix update or tensor-network-based simulations. We focus our comparison with the latter category of simulations, with the note that stabilizer-based simulation techniques are more scalable for a rough understanding of noises that can be formulated as Pauli errors. Due to the prohibitively large density matrix involved in a 17-qubit simulation (2^{34} entries in the worst case), most of the simulation efforts adopt sampling-based simulation techniques [20, 33], where by "sampling-based" we refer to methods that repeatedly sample from the underlying distribution of the syndrome outcomes and perform simulations conditioned on the particular syndrome.

Although each trial may take a relatively short time, sampling-based approaches usually suffer from the large number of random trials needed in order to reduce the variance and obtain a sufficiently accurate estimation. On the other hand, exact simulation methods, such as tensor-network-based approaches, take significantly longer time for one trial but do not need repeated experiments. It is not clear *a priori* which simulation method one should adopt, but we present here a simple argument that tensor-network-based simulation is more feasible in our regime of interest.

Suppose that each random trial involves a full density-matrix simulation, which takes time exponential with respect to the number of qubits in the system. In our noise model, it suffices to consider at most 13 qubits at the same time, and the cost for running one trial is roughly 2^{26} basic arithmetic operations. To achieve an accuracy of 10^{-5} for the logical channel entries, one needs about $10^{10} \approx 2^{33}$ trials, resulting in a total running time of 2^{59}. A tensor-network based approach, however, runs in time exponential with respect to the treewidth of the tensor network. For the same noise model, with 2+1 syndrome extraction rounds, the resulting tensor network has a treewidth upper bounded by 38. The tensor network-based simulation thus takes time 2^{38}, over one million times more efficient than sampling-based approaches.

Of course, such comparisons depend heavily on the specifics of the experiment to be conducted, and there could be cases where sampling-based techniques are feasible while exact computation is not. Nevertheless, tensor-network-based simulation allows us to simulate a physically well-motivated model in the presence of crosstalk.

Our tensor network-based simulation framework clearly demonstrates the capability and flexibility of tensor network-based approaches in dealing with large-scale problems that do not necessarily lie in the scope of quantum circuits, in that the exact

logical channel can be computed in merely one hour on a single GPU. Without much modification, our tensor network contraction schemes can be applied to quantum memory experiments with different gate scheduling, different noise parameters, and even slight variants of the Surface-17 based on the newly proposed XZZX codes, etc.

It is undeniable that the particular way of studying the performance of the optimal decoder is hard to scale up due to overwhelmingly large final tensor. However, tensor network-based approaches paves a new way towards the study of quantum error correction through exact simulation. There are many ways to get around the limitation of big final result likely induced by the optimal decoder, including a suboptimal decoder that fits better in the tensor network framework, or an approximate contraction algorithm that extracts the property of the final tensor network without writing it down explicitly. We leave these possible extensions to future work.

---

[1] Igor L. Markov and Yaoyun Shi. Simulating quantum computation by contracting tensor networks. *SIAM Journal on Computing*, 38(3):963–981, 2008.

[2] Johnnie Gray and Stefanos Kourtis. Hyper-optimized tensor network contraction. *Quantum*, 5:410, March 2021.

[3] Vibhav Gogate and Rina Dechter. A complete anytime algorithm for treewidth. *arXiv preprint arXiv:1207.4109*, 2012.

[4] Hisao Tamaki. Positive-instance driven dynamic programming for treewidth, 2019. Parameterized Algorithms and Computational Experiments Challenge, https://pacechallenge.org/.

[5] Roman Schutski, Danil Lykov, and Ivan Oseledets. Adaptive algorithm for quantum circuit simulation. *Physical Review A*, 101(4):042335, 2020.

[6] Danylo Lykov, Roman Schutski, Alexey Galda, Valerii Vinokur, and Yurii Alexeev. Tensor network quantum simulator with step-dependent parallelization. *arXiv preprint arXiv:2012.02430*, 2020.

[7] Frank Arute, Kunal Arya, Ryan Babbush, Dave Bacon, Joseph C Bardin, Rami Barends, Rupak Biswas, Sergio Boixo, Fernando GSL Brandão, David A Buell, et al. Quantum supremacy using a programmable superconducting processor. *arXiv preprint arXiv:1910.11333*, 2019. Data retrieved from datadryad.org, `https://datadryad.org/stash/dataset/doi:10.5061/dryad.k6t1rj8`.

[8] Benjamin Villalonga, Sergio Boixo, Bron Nelson, Christopher Henze, Eleanor Rieffel, Rupak Biswas, and Salvatore Mandrà. A flexible high-performance simulator for verifying and benchmarking quantum circuits implemented on real hardware. *npj Quantum Information*, 5:1–16, 2019.

[9] Frank Arute, Kunal Arya, Ryan Babbush, Dave Bacon, Joseph C Bardin, Rami Barends, Rupak Biswas, Sergio Boixo, Fernando GSL Brandão, David A Buell, et al. Quantum supremacy using a programmable superconducting processor. *Nature*, 574(7779):505–510, 2019.

[10] Edwin Pednault, John A Gunnels, Giacomo Nannicini, Lior Horesh, and Robert Wisnieff. Leveraging secondary storage to simulate deep 54-qubit sycamore circuits. *arXiv preprint arXiv:1910.09534*, 2019.

[11] Thomas Häner and Damian S Steiger. 0.5 petabyte simulation of a 45-qubit quantum circuit. In *Proceedings of the International Conference for High Performance Computing, Networking, Storage and Analysis*, pages 1–10, 2017.

[12] Sergio Boixo, Sergei V Isakov, Vadim N Smelyanskiy, Ryan Babbush, Nan Ding, Zhang Jiang, Michael J Bremner, John M Martinis, and Hartmut Neven. Characterizing quantum supremacy in near-term devices. *Nature Physics*, 14(6):595–600, 2018.

[13] Igor L Markov, Aneeqa Fatima, Sergei V Isakov, and Sergio Boixo. Quantum supremacy is both closer and farther than it appears. *arXiv preprint arXiv:1807.10749*, 2018.

[14] Feng Pan and Pan Zhang. Simulating the sycamore quantum supremacy circuits. *arXiv preprint arXiv:2103.03074*, 2021.

[15] Boaz Barak, Chi-Ning Chou, and Xun Gao. Spoofing linear cross-entropy benchmarking in shallow quantum circuits. *arXiv preprint arXiv:2005.02421*, 2020.

[16] Edward Farhi, Jeffrey Goldstone, and Sam Gutmann. A quantum approximate optimization algorithm. *arXiv preprint arXiv:1411.4028*, 2014.

[17] Mario Szegedy. What do QAOA energies reveal about graphs? *arXiv preprint arXiv:1912.12277*, 2019.

[18] László Babai. Graph isomorphism in quasipolynomial time [extended abstract]. In *Proceedings of the 48th Annual ACM SIGACT Symposium on Theory of Computing, STOC 2016, Cambridge, MA, USA, June 18-21, 2016*, pages 684–697, 2016.

[19] Kamil Bradler, Shmuel Friedland, Josh Izaac, Nathan Killoran, and Daiqin Su. Graph isomorphism and gaussian boson sampling, 2018.

[20] TE O'Brien, B Tarasinski, and L DiCarlo. Density-matrix simulation of small surface codes under current and projected experimental noise. *npj Quantum Information*, 3(1):1–8, 2017.

[21] J Pablo Bonilla Ataides, David K Tuckett, Stephen D Bartlett, Steven T Flammia, and Benjamin J Brown. The xzzx surface code. *Nature communications*, 12(1):1–12, 2021.

[22] Daniel A. Lidar and Todd A. Brun. *Quantum Error Correction*. Cambridge University Press, 2013.

[23] Eric Dennis, Alexei Kitaev, Andrew Landahl, and John Preskill. Topological quantum memory. *Journal of Mathematical Physics*, 43(9):4452–4505, 2002.

[24] Austin G Fowler, Matteo Mariantoni, John M Martinis, and Andrew N Cleland. Surface codes: Towards practical large-scale quantum computation. *Physical Review A*, 86(3):032324, 2012.

[25] Nicolas Delfosse and Gilles Zémor. Linear-time maximum likelihood decoding of surface codes over the quantum erasure channel. *Physical Review Research*, 2(3):033042, 2020.

[26] Nicolas Delfosse and Naomi H Nickerson. Almost-linear time decoding algorithm for topological codes. *arXiv preprint arXiv:1709.06218*, 2017.

[27] Muyuan Li, Daniel Miller, Michael Newman, Yukai Wu, and Kenneth R Brown. 2d compass codes. *Physical Review X*, 9(2):021041,

2019.
[28] Shilin Huang and Kenneth R Brown. Fault-tolerant compass codes. *Physical Review A*, 101(4):042312, 2020.
[29] Poulami Das, Christopher A Pattison, Srilatha Manne, Douglas Carmean, Krysta Svore, Moinuddin Qureshi, and Nicolas Delfosse. A scalable decoder micro-architecture for fault-tolerant quantum computing. *arXiv preprint arXiv:2001.06598*, 2020.
[30] MA Rol, F Battistel, FK Malinowski, CC Bultink, BM Tarasinski, R Vollmer, N Haider, N Muthusubramanian, A Bruno, BM Terhal, et al. Fast, high-fidelity conditional-phase gate exploiting leakage interference in weakly anharmonic superconducting qubits. *Physical Review Letters*, 123(12):120502, 2019.
[31] Dripto M Debroy, Muyuan Li, Michael Newman, and Kenneth R Brown. Stabilizer slicing: Coherent error cancellations in low-density parity-check stabilizer codes. *Physical Review Letters*, 121(25):250502, 2018.
[32] Leonardo DiCarlo, Jerry M Chow, Jay M Gambetta, Lev S Bishop, Blake R Johnson, DI Schuster, J Majer, Alexandre Blais, Luigi Frunzio, SM Girvin, et al. Demonstration of two-qubit algorithms with a superconducting quantum processor. *Nature*, 460(7252):240–244, 2009.
[33] Yu Tomita and Krysta M Svore. Low-distance surface codes under realistic quantum noise. *Physical Review A*, 90(6):062320, 2014.
[34] Andrew S Darmawan and David Poulin. Tensor-network simulations of the surface code under realistic noise. *Physical Review Letters*, 119(4):040502, 2017.
[35] Colin J Trout, Muyuan Li, Mauricio Gutiérrez, Yukai Wu, Sheng-Tao Wang, Luming Duan, and Kenneth R Brown. Simulating the performance of a distance-3 surface code in a linear ion trap. *New Journal of Physics*, 20(4):043038, 2018.
[36] Sergey Bravyi, Matthias Englbrecht, Robert König, and Nolan Peard. Correcting coherent errors with surface codes. *npj Quantum Information*, 4(1):1–6, 2018.
[37] As of the time of conducting the comparison, the latest Github version is 949635c6783435fc384553ec28c7c038dc786e01
[38] https://github.com/alibaba/acqdp
